# 2*E*,4*E*-Decadienoic Acid, a Novel Anti-Oomycete Agent from Coculture of Bacillus subtilis and Trichoderma asperellum

**DOI:** 10.1128/spectrum.01542-22

**Published:** 2022-08-09

**Authors:** Xi-Fen Zhang, Qing-Yu Li, Mei Wang, Si-Qi Ma, Yan-Fen Zheng, Yi-Qiang Li, Dong-Lin Zhao, Cheng-Sheng Zhang

**Affiliations:** a Tobacco Research Institute of Chinese Academy of Agricultural Sciences, Qingdao, People’s Republic of China; State Key Laboratory of Microbial Resources, Institute of Microbiology, Chinese Academy of Sciences

**Keywords:** metabolomics, transcriptomics, oxidative stress, cell membrane, mitochondria

## Abstract

Phytophthora nicotianae is an oomycete pathogen of global significance threatening many important crops. It is mainly controlled by chemosynthetic fungicides, which endangers ecosystem and human health; thus, there is an urgent need to explore alternatives for these fungicides. In this study, a new anti-oomycete aliphatic compound, 2*E*,4*E-*decadienoic acid (DDA), was obtained through coculture of Bacillus subtilis Tpb55 and Trichoderma asperellum HG1. Both *in vitro* and *in vivo* tests showed that DDA had a strong inhibitory effect against *P. nicotianae*. In addition, rhizosphere microbiome analysis showed that DDA reduced the relative abundance of Oomycota in rhizosphere soil. Transcriptome sequencing (RNA-Seq) analysis revealed that treatment of *P. nicotianae* with DDA resulted in significant downregulation of antioxidant activity and energy metabolism, including antioxidant enzymes and ATP generation, and upregulation of membrane-destabilizing activity, such as phospholipid synthesis and degradation. The metabolomic analysis results implied that the pathways influenced by DDA were mainly related to carbohydrate metabolism, energy metabolism, and the cell membrane. The biophysical tests further indicated that DDA produced oxidative stress on *P. nicotianae*, inhibited antioxidant enzyme and ATPase activity, and increased cell membrane permeability. Overall, DDA exerts inhibitory activity by acting on multiple targets in *P. nicotianae*, especially on the cell membrane and mitochondria, and can therefore serve as a novel environment-friendly agent for controlling crop oomycete disease.

**IMPORTANCE** *P*. *nicotianae* is an oomycete pathogen that is destructive to crops. Although some oomycete inhibitors have been used during crop production, most are harmful to the ecology and lead to pathogen resistance. Alternatively, medium-chain fatty acids have been reported to exhibit antimicrobial activity in the medical field in previous studies; however, their potential as biocontrol agents has rarely been evaluated. Our *in vivo* and *in vitro* analyses revealed that the medium-chain fatty acid 2*E*,4*E-*decadienoic acid (DDA) displayed specific inhibitory activity against oomycetes. Further analysis indicated that DDA may acted on multiple targets in *P*. *nicotianae*, especially on the cell membrane and mitochondria. Our findings highlight the potential of DDA in controlling oomycete diseases. In conclusion, these results provide insights regarding the future use of green and environment-friendly anti-oomycete natural products for the prevention and control of crop oomycete diseases.

## INTRODUCTION

Oomycota is a phylum of fungus-like organisms in the clade Stramenopile (1). More than 1,800 oomycete species have been reported, including many important plant pathogens, notably those of the genus *Phytophthora*, which infect many crucial crops and cause various devastating diseases. Although oomycetes are morphologically similar to fungi, they differ with regard to metabolism, cell wall composition, and reproduction mode (2), resulting in the ineffectiveness of fungicides that target chitin. Currently, fungicides with anti-oomycete activity usually contain tin, copper, or other persistent chemicals, resulting in the increase of negative effects such as pesticide residues, environmental pollution, and pathogen resistance and putting the ecosystem and human health at risk (3). Consequently, it is urgent to develop environmentally friendly technologies to control oomycotal phytopathogens.

One strategy to alleviate this problem is to seek new anti-oomycete natural products, including those that have already been approved or are regarded as safe antimicrobial agents, such as fatty acids (4). Some fatty acids, especially medium-chain fatty acids (MCFAs) with a carbon chain length of 6 to 12 carbon atoms, have been reported to exhibit antimicrobial activity in previous research. For example, heptanoic, octanoic, nonanoic, decanoic, undecanoic, and lauric acids effectively inhibited the hyphal growth of Candida albicans (5). Fatty acids exert their antimicrobial effects by targeting different cellular functions, including protein synthesis, fatty acid metabolism, morphogenesis, and biofilm formation (5–7). To date, studies on the applications of MCFAs have focused mainly on clinical and veterinary research; however, the potential of MCFAs as agents for crop disease prevention has rarely been evaluated. In addition, although the inhibitory activities of MCFAs against fungi and bacteria have been demonstrated, their anti-oomycete ability and modes of action are yet unknown. In particular, the MCFA 2*E*,4*E*-decadienoic acid (DDA) was first identified from stillingia oil in Sapium sebiferum seeds (8). Although some natural antimicrobial products containing DDA residues in their structures have been discovered, there are no reports on the anti-oomycete activity of DDA.

Phytophthora nicotianae is an important representative species of *Phytophthora* which causes numerous destructive crop diseases such as tobacco black shank. *P. nicotianae* has a wide host range and strong transmission power, infecting 255 plant species in 90 families, including many economically important crops, such as tobacco, tomato, and citrus (9). In our previous study (10), we showed that the inhibitory activity of a fermentation broth filtrate of Trichoderma asperellum HG1 and Bacillus subtilis Tpb55 on *P. nicotianae* was significantly stronger than that of single culture of either strain. In the present study, we obtained DDA, shown to be of microbial origin, through coculture of B. subtilis Tpb55 and *T. asperellum* HG1; moreover, preliminary experiments showed that DDA had strong inhibitory activity on *P*. *nicotianae*.

To evaluate its potential as a bioactive agent, in this study, we synthesized DDA and evaluated its antimicrobial activity against common plant-pathogenic fungi. Following microscopic observation of the effect of DDA on *P. nicotianae* mycelia, we verified the inhibitory activity against *P. nicotianae* in plants using pot experiments and analyzed the effect on the oomycete community in rhizosphere soil via high-throughput analysis. Moreover, transcriptome and metabolome analyses were used to reveal the target cellular functions to better understand the mechanism of DDA activity against the oomycete pathogen *P. nicotianae*. Furthermore, the physiological response of *P. nicotianae* to DDA exposure was also evaluated. Our findings are expected to promote the sustainable control of oomycete phytopathogens and provide a better mechanistic insight into the anti-oomycete effects of MCFAs.

## RESULTS

### Growth inhibition of DDA against oomycetes and fungi.

By tracing the inhibitory activity of *P. nicotianae*, a principal bioactive component (fraction 3) was purified from the extract (fractions 1 to 7) of the coculture fermentation broth of *T. asperellum* HG1 and B. subtilis Tpb55 (see Table S1 in the supplemental material) and identified as DDA based on the nuclear magnetic resonance (NMR) and mass spectrometry (MS) spectra (8) (Fig. S1 to S3). Furthermore, we found that DDA was produced by *T. asperellum* HG1 rather than B. subtilis Tpb55 using high-performance liquid chromatography (HPLC); nevertheless, the yield of DDA from coculture of these two strains was higher than that from single cultures, consistent with our prior results regarding inhibitory activity (10). However, because of the low yields of DDA, it was subsequently synthesized from 2*E*,4*E*-deca-2,4-dienal for further experiments. DDA had a strong inhibitory effect on *P. nicotianae* mycelial growth, with a 50% effective concentration (EC_50_) of 34.59 μg/mL, which increased dose dependently (Fig. S4). Furthermore, the activities of DDA against other oomycetes (Phytophthora capsici, Phytophthora sojae, Pythium aphanidermatum, Pythium coloratum, and Pythium ultimum) and six phytopathogenic fungi (Alternaria alternata, Botrytis cinerea, Fusarium graminearum, Ceratobasidium cornigerum, Magnaporthe oryzae, and Physalospora piricola Nose) were also evaluated, as shown in Table 1. At a 100-μg/mL concentration, DDA exhibited strong anti-oomycete activity while displaying weak antifungal activity, indicating that DDA has specific activity against oomycetes.

**TABLE 1:** Inhibition rates of DDA (100 μg/mL) on different phytopathogens

Phytopathogen	Plant disease	Inhibition rate (%)^*a*^
Alternaria alternata	Tobacco brown spot	6.120 ± 1.56 h
Botrytis cinerea	Grape gray mold	40.57 ± 1.87 f
Fusarium graminearum	Fusarium head blight of wheat	20.44 ± 2.01 g
*Ceratobasidium cornigerum*	Wheat sharp eyespot	51.63 ± 2.84 e
Magnaporthe oryzae	Rice blast	18.60 ± 0.47 g
*Physalospora piricola* Nose	Apple ring rot	8.460 ± 0.44 h
*Pythium aphanidermatum*	Damping-off or root or fruit rot of plants	82.68 ± 0.67 c
*Pythium coloratum*	Root rot of cruciferous plants	77.18 ± 0.34 d
*Pythium ultimum*	Damping-off or root rot of plants	92.59 ± 0.15 b
*Phytophthora capsici*	*Phytophthora* blight of pepper	94.21 ± 0.37 a
*Phytophthora sojae*	*Phytophthora* root rot of soybean	97.25 ± 0.60 a
*Phytophthora nicotianae*	Tobacco black shank	83.07 ± 0.20 c

a Significant differences between different pathogens at the level of a *P* value of <0.05 are indicated by different lowercase letters.

### Effects of DDA on morphology and ultrastructure of *P. nicotianae*.

**FIG 1:**
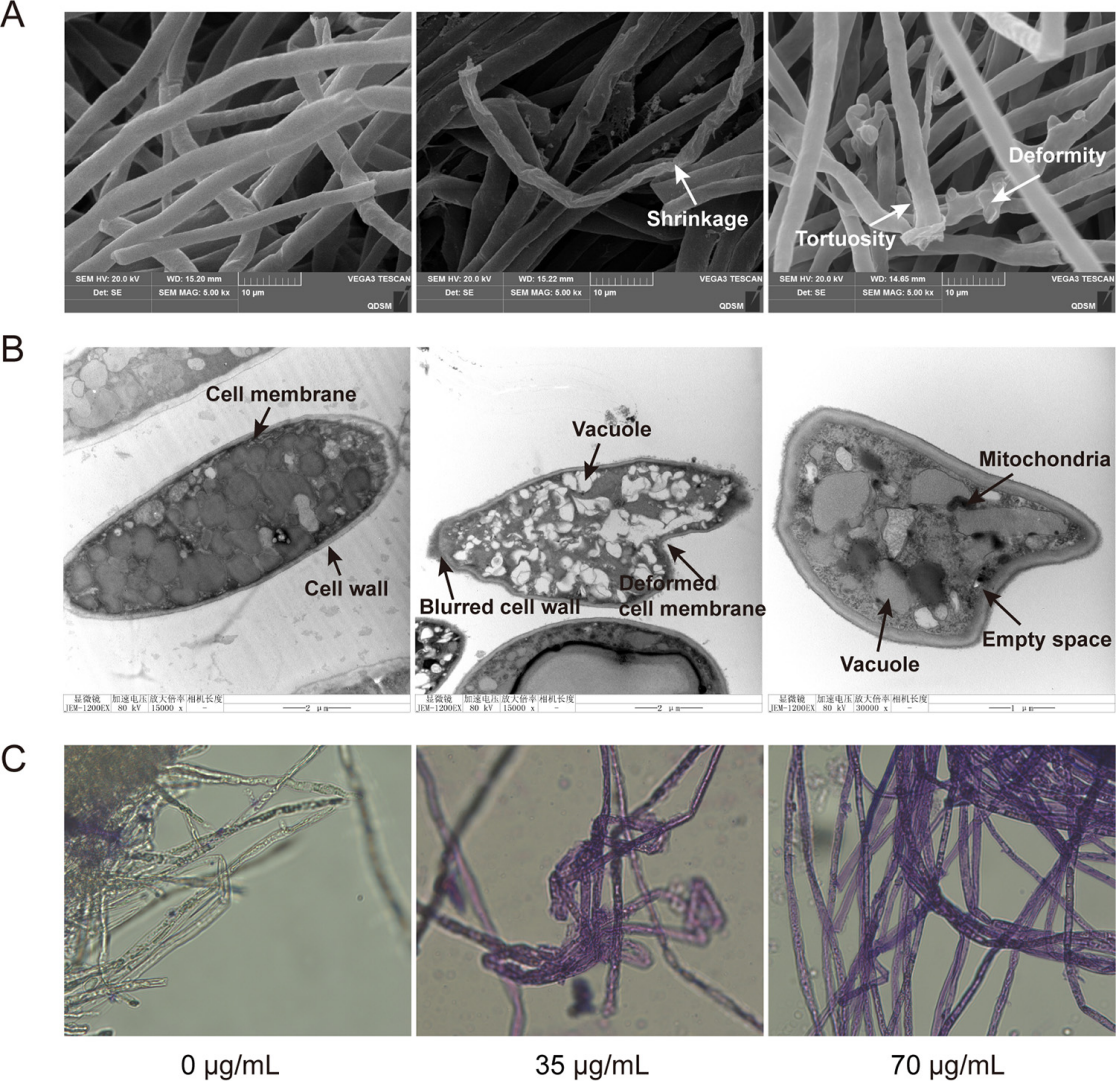
Effects of DDA on the morphology of *P. nicotianae*. (A) SEM images of the morphology of *P. nicotianae* (EC_50_ = 35 μg/mL and 2-fold EC_50_ = 70 μg/mL). Magnification, ×5,000. Bar = 10 μm). (B) TEM images of the ultrastructure of *P. nicotianae*. Magnifications, ×25,000 (left), ×15,000 (center), and ×30,000 (right). Bars = 2 μm (left and center) and 1 μm (right). (C) Tannic acid staining observation of cell wall integrity. Magnification, ×400.

The effects of DDA on the mycelial morphology and cellular ultrastructure of *P. nicotianae* were observed using scanning electron microscopy (SEM) (Fig. 1A) and transmission electron microscopy (TEM) (Fig. 1B). As depicted in Fig. 1A, the mycelia of control group were normal, uniform, smooth, and round, whereas the mycelia exposed to DDA were bent, shrunken, collapsed, and deformed. TEM observation showed that all organelles in the control group were arranged regularly and the cell walls were clear (Fig. 1B). However, some changes were observed in the DDA treatment group, including cell deformation, fuzzy damage of cell wall and membrane, unequal vacuole size, and blurred organelles. The results demonstrated that the cell wall and membrane were significantly affected by DDA.

Tannic acid is an acidic mordant which can cause the cell wall to form a colorable complex. After tannic acid staining, the cytoplasm with an intact cell wall was colorless or light purple, whereas the cells with damaged cell walls were dyed dark purple (11). As seen in Fig. 1C, the mycelial cytoplasm of the group without DDA was colorless or dyed light purple. However, those treated with DDA were strongly stained and dyed dark purple, suggesting that DDA could destroy the cell wall integrity of *P. nicotianae*.

### Effects of DDA on tobacco black shank under greenhouse conditions.

**FIG 2:**
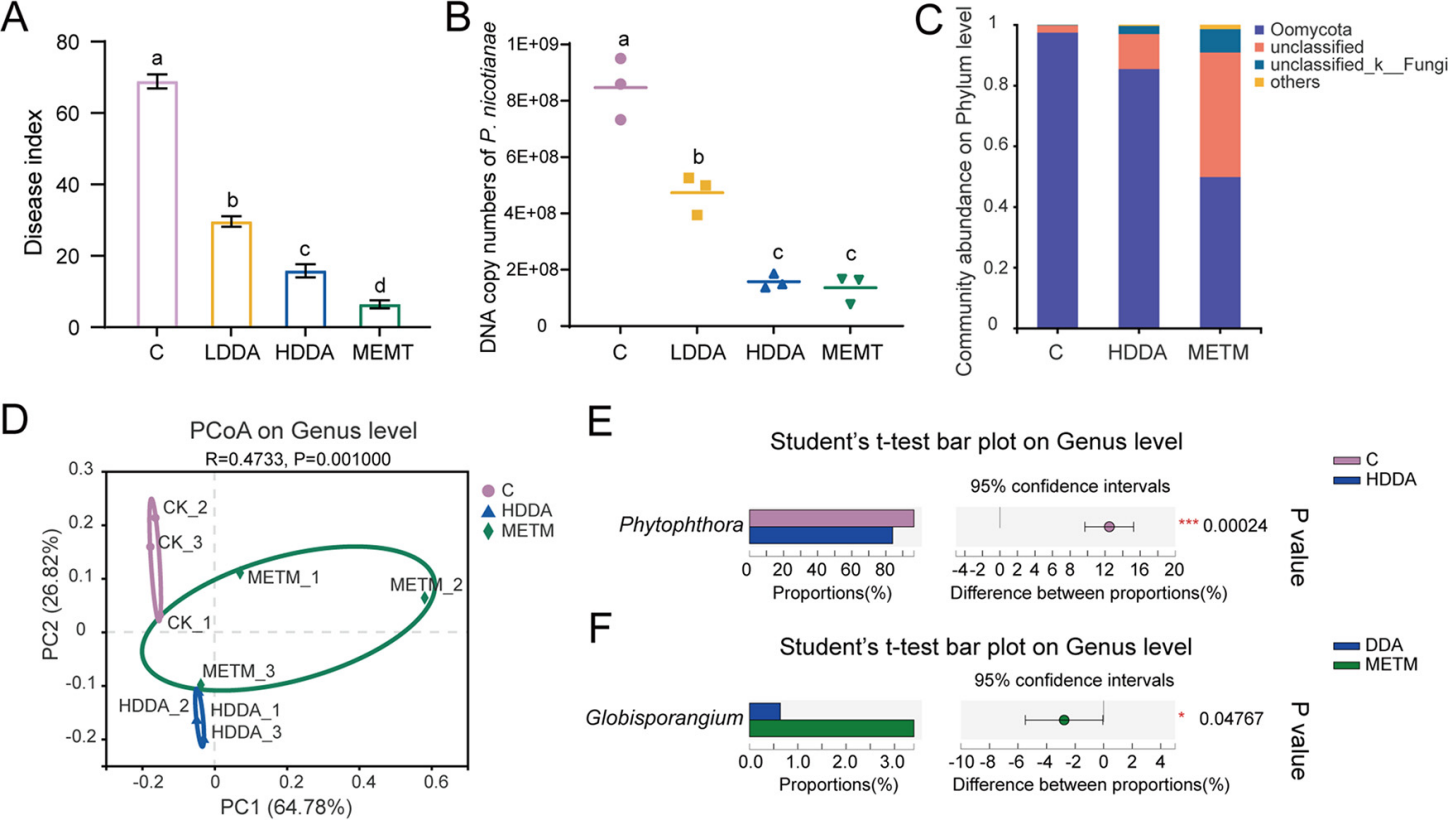
Effects of DDA on disease control and composition of the oomycete community in rhizosphere soil. (A) Disease index of tobacco black shank in the pot experiment. (B) DNA copy numbers of *P. nicotianae* in rhizosphere soil. Different lowercase letters represent significant differences between groups (*P* < 0.05) (A and B). (C) Percentage of community abundance of oomycetes at the phylum level. (D) PCoA analysis of oomycetes at the genus level. (E) Significantly different genera of oomycetes at the genus level between the C and HDDA groups. (F) Significantly different genera of oomycetes at the genus level between the HDDA and METM groups. *, *P* < 0.05; **, *P* < 0.01; ***, *P* < 0.001.

To verify the inhibition of *P. nicotianae* by DDA *in vivo*, a pot experiment was carried out in the greenhouse, including four treatments: a group inoculated with *P. nicotianae* only (C), a group inoculated with *P. nicotianae* and a 1,600-fold dilution of DDA (LDDA), a group inoculated with *P. nicotianae* and an 800-fold dilution of DDA (HDDA), and a group inoculated with *P. nicotianae* and an 800-fold dilution of metalaxyl-M (METM) (a widely used germicide for oomycetes, used as the positive control). The disease index of DDA against tobacco black shank is shown in Fig. 2A. On the 7th day after treatment, the disease indexes in the treatment groups (5.19 to 31.11) were significantly lower than those of the control group (68.15 to 71.11). The disease index of tobacco black shank decreased to 56.99% and 77.06% in the LDDA and HDDA groups, respectively, which positively correlated with the DDA concentration. Moreover, the results of real-time quantitative PCR (qPCR) of *P. nicotianae* in the rhizosphere soil of tobacco showed that the DNA copy numbers of the pathogen in the LDDA, HDDA, and METM groups significantly decreased compared to those in the control group (C) (Fig. 2B), indicating that DDA diminished the colonization ability of *P. nicotianae* in the rhizosphere and had good activity against tobacco black shank.

### Effects of DDA on composition of the oomycete community in rhizosphere soil.

To detect the effects of HDDA and METM on soil oomycete community, high-throughput sequencing analysis was carried out on rhizosphere soil samples with specific oomycete primers. A total of 2,118,512 sequences were obtained, which were aggregated into 2,577 operational taxonomic units (OTUs). All OTUs were divided into 26 phyla, of which the dominant phylum was Oomycota, accounting for 97.44%, 85.40%, and 49.49% in the C, HDDA, and METM treatment groups, respectively (Fig. 2C). Notably, compared with that in the control group, the relative abundance of Oomycota in the HDDA and METM groups decreased by 12.4% and 47.65%, respectively, which were consistent with the disease index of tobacco black shank. In addition, the result of principal-coordinate analysis (PCoA) based on Bray-Curtis distance showed that C, HDDA, and METM groups formed different clusters at the genus level (Fig. 2D), suggesting that DDA significantly changed the composition of the oomycetal community at the genus level in the rhizosphere soil compared with that of the control.

At the genus level, significant differences were observed between HDDA and C and between HDDA and METM. The abundances of *Phytophthora* were at high levels in all the three groups, which may have been caused by the inoculation of *P. nicotianae* in the soil. There were 25 OTUs detected in genus *Phytophthora*, all of which were *Phytophthora nicotianae*, accounting for 99.22%, 98.28%, and 96.92% of the Oomycota detected in C, HDDA, and METM, respectively. A dramatically lower abundance of *Phytophthora* was observed in the HDDA group than that in the control group (Fig. 2E). However, *Phytophthora* levels did not differ significantly between the HDDA and METM groups. In addition, the abundance of *Globisporangium* in the HDDA group was lower than that in the METM group, whereas other common plant-pathogenic oomycetes showed no significant difference in these two groups (Fig. 2F).

### RNA-Seq analysis of *P. nicotianae* with DDA treatment.

**FIG 3:**
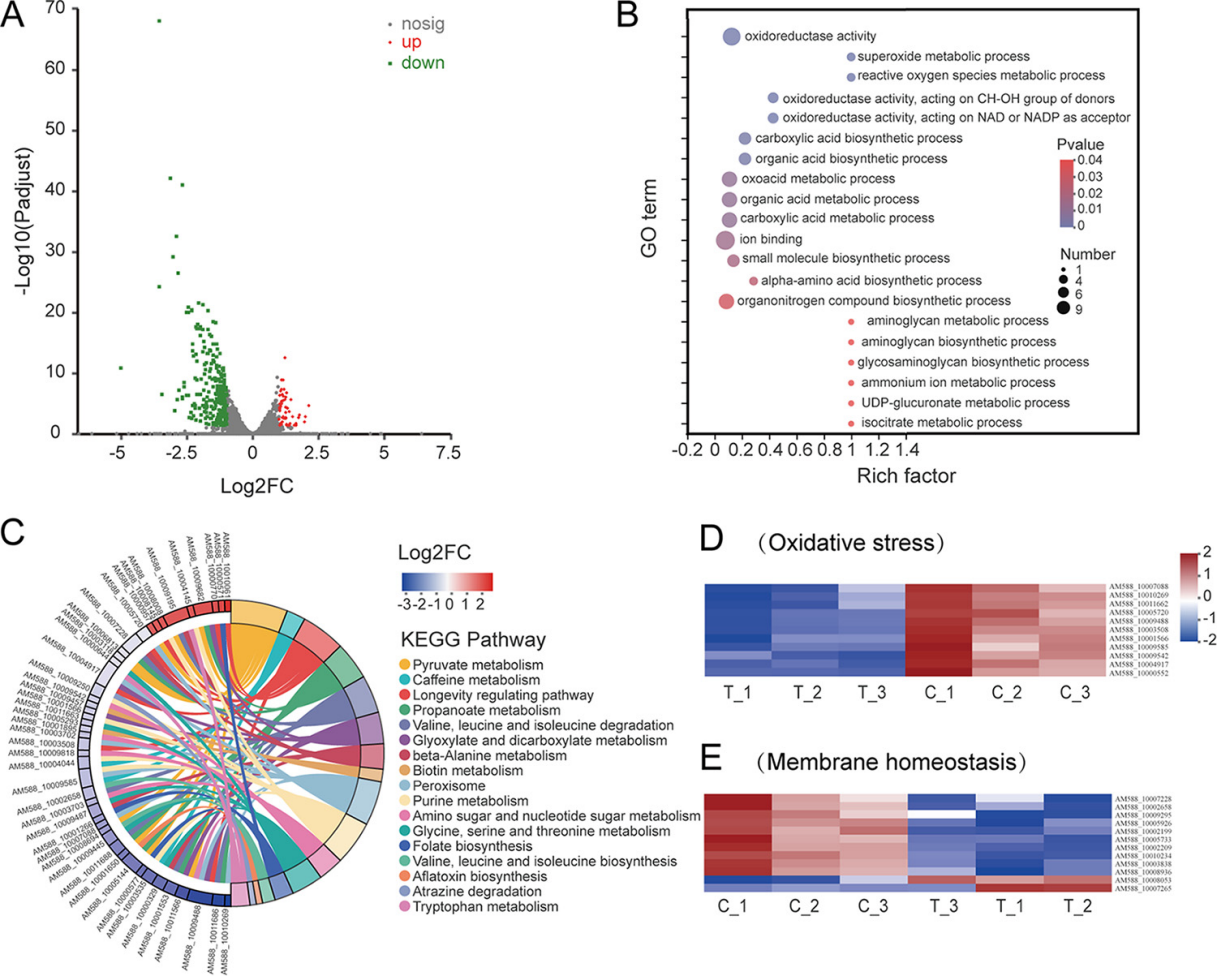
RNA-Seq analysis of *P. nicotianae* exposure to DDA. (A) Volcano plot of DEGs. Red dots represent upregulated DEGs, whereas green dots represent downregulated DEGs; gray dots represent genes with no significant difference. (B) GO enrichment analysis. See Table S3 for GO terms. (C) KEGG enrichment analysis. The colored bands on the right indicate the KEGG pathways, and the colored bands on the left indicate the DEGs (Table S4). (D) Major DEGs related to oxidative stress affected in *P. nicotianae* exposed to DDA compared to untreated groups (Table S7). (E) Major DEGs related to membrane homeostasis affected in *P. nicotianae* exposed to DDA compared to untreated groups (Table S8).

To explore the effect of DDA on the gene expressions of *P. nicotianae*, we performed transcriptome sequencing (RNA-Seq) to identify the significant differentially expressed genes (DEGs) caused by DDA-induced stress. Reads were filtered following transcriptome sequencing, yielding 40.33 Gb of clean data, with the percentage of Q30 bases being over 94.53% (Table S2). A total of 9,450 expressed genes were detected, all representing known genes. Compared with the control, 317 DEGs were found in the DDA treatment group, among which 54 were upregulated and 263 were downregulated (Fig. 3A). The classification of DEGs is shown in Fig. S5.

### Validation of RNA-Seq data.

To verify the expression of DEGs determined using RNA-Seq, we randomly selected 10 genes to undergo real-time quantitative reverse transcription-PCR (qRT-PCR) analysis for estimation of their expression levels following exposure to DDA. The changes of the 10 genes as determined by qRT-PCR were consistent with those of the RNA-Seq analyses, with three being upregulated and seven downregulated (Fig. S6), indicating that our RNA-Seq data were dependable.

### Enrichment analysis of DEGs.

Based on homology search, a total of 109 DEGs were annotated to the Gene Ontology (GO) database. The top 20 ranked GO terms (*P* < 0.05) are listed in Fig. 3B and Table S3 in view of GO enrichment analysis with 109 DEGs. Among these, the most significant enrichment term was “superoxide metabolic process,” followed by “reactive oxygen species metabolic process,” and “oxidoreductase activity.” These results suggested that the accumulation of reactive oxygen species (ROS) in *P. nicotianae* may be affected by DDA-induced stress.

Compared with the control group, 125 DEGs associated with treatment group were annotated to the Kyoto Encyclopedia of Genes and Genomes (KEGG) database and mapped to 17 KEGG pathways (*P* < 0.05). The highly KEGG-enriched pathways included “pyruvate metabolism” (map00620), “longevity regulating pathway-multiple species” (map04213), “valine, leucine, and isoleucine degradation” (map00280), “glyoxylate and dicarboxylate metabolism” (map00630), “peroxisome” (map04146), and “purine metabolism” (map00230) (Fig. 3C; Table S4). Previous studies have shown that these pathways were related to energy and substance metabolism, oxidation-reduction processes, and cell membranes (3, 12, 13).

Moreover, we observed that DEGs related to oxidative stress, cell membrane homeostasis, and substance and energy metabolism were significantly downregulated. The main DEGs related to oxidative stress and membrane homeostasis were then analyzed using cluster analysis (Fig. 3D and E). Compared to the control, DEGs encoding peroxidase and antioxidant enzymes such as AM588_10011662, AM588_10003508, and AM588_10009488 were downregulated in the treatment group (Table S5). Similarly, the DEGs encoding enzymes involved in cell membrane component synthesis (AM588_10009295 and AM588_10008936) and transporters (AM588_10003838 and AM588_10010234) were also downregulated (Table S6). In addition, DEGs involved in substance and energy metabolism were significantly downregulated, such as malate dehydrogenase (AM588_10008694), pyruvate carboxylase (AM588_10001650), and isocitrate dehydrogenase (AM588_10010269) (Table S7). These downregulated DEGs confirmed that DDA inhibited the activities of peroxidase and antioxidant enzymes, likely causing a large accumulation of ROS, which then destroyed the cell membrane permeability, hindering the metabolism of the organism.

### Metabonomic analysis of *P. nicotianae* with DDA treatment.

**FIG 4:**
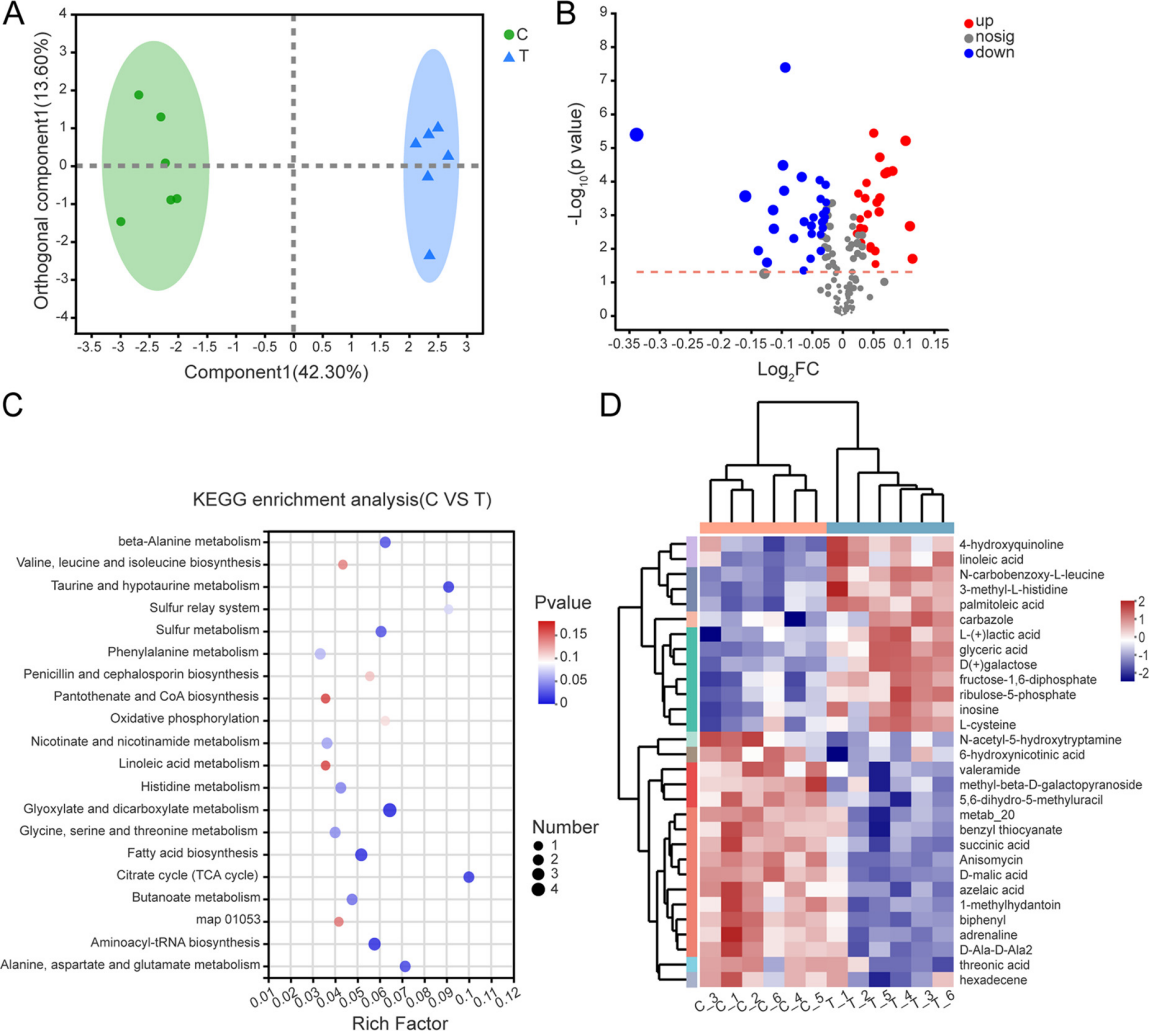
Metabolomic analysis of *P. nicotianae* exposure to DDA. (A) OPLS-DA model of *P. nicotianae* samples with DDA treatment and the control without DDA. (B) Volcano plot of DEMs. Red dots represent upregulated DEMs, whereas blue dots represent downregulated DEMs; gray dots represent genes with no significant difference. (C) KEGG enrichment analysis of DEMs. map01053, biosynthesis of siderophore group nonribosomal peptides. (D) Heat map of significantly differential metabolites of *P. nicotianae* following exposure to DDA. The *x* axis represents treatments, whereas the *y* axis represents DEMs. metab_20, 12a-hydroxy-9-demethylmunduserone-8-carboxylic acid.

To better understand the mode of action of DDA against *P. nicotianae*, gas chromatography-mass spectrometry (GC-MS) was used to analyze the metabolomics of *P. nicotianae* treated with DDA, which detected 152 metabolites (Table S8). Orthogonal partial least-squares discriminant analysis (OPLS-DA) showed that the DDA and control groups were well separated, revealing that the metabolism of *P. nicotianae* was significantly affected by DDA (Fig. 4A). A total of 54 differentially expressed metabolites (DEMs) were screened using a VIP (variable important in projection) value of >1 and a *P* value of <0.05, inclusive of 25 upregulated metabolites, and 29 downregulated metabolites (Fig. 4B; Table S9).

KEGG enrichment analysis demonstrated that the following *P. nicotianae* pathways changed significantly following DDA treatment: “citrate cycle (TCA cycle),” “taurine and hypotaurine metabolism,” “glyoxylate and dicarboxylate metabolism,” “alanine, aspartate and glutamate metabolism,” “aminoacyl-tRNA biosynthesis,” and “fatty acid biosynthesis” (Fig. 4C; Table S10). Organic, amino, and nucleic acids in the DDA treatment changed markedly compared to the control. Succinic, malic, and citric acids involved in substance and energy metabolism pathways (e.g., the tricarboxylic acid [TCA] cycle and oxidative phosphorylation) were downregulated. Conversely, l-methionine and l-cysteine involved in amino acid metabolism, glyceric, myristic, palmitoleic, and linoleic acids involved in lipid metabolism, and inosine and adenosine involved in purine metabolism were prominently upregulated. This may be related to the massive accumulation of ROS, which can attack biological macromolecules such as DNA, proteins, and lipids in organisms. The results demonstrated that DDA interfered with the steady-state metabolism of organic acids, amino acids, lipids, nucleic acids, and carbohydrates in *P. nicotianae* (Fig. 4D).

### Effects of DDA on oxidative stress of *P. nicotianae*.

The alterations in the above pathways suggested that carbohydrate metabolism, energy metabolism, cell membrane, and ROS metabolism might be affected by DDA, consistent with the transcriptomic analysis results. Based on this consideration, we examined the effects of DDA on cell membrane integrity and permeability, ROS, superoxide dismutase (SOD) activity, ATPase activity, ATP content, and citric acid content to verify the results of transcriptomics and metabolomics.

**FIG 5:**
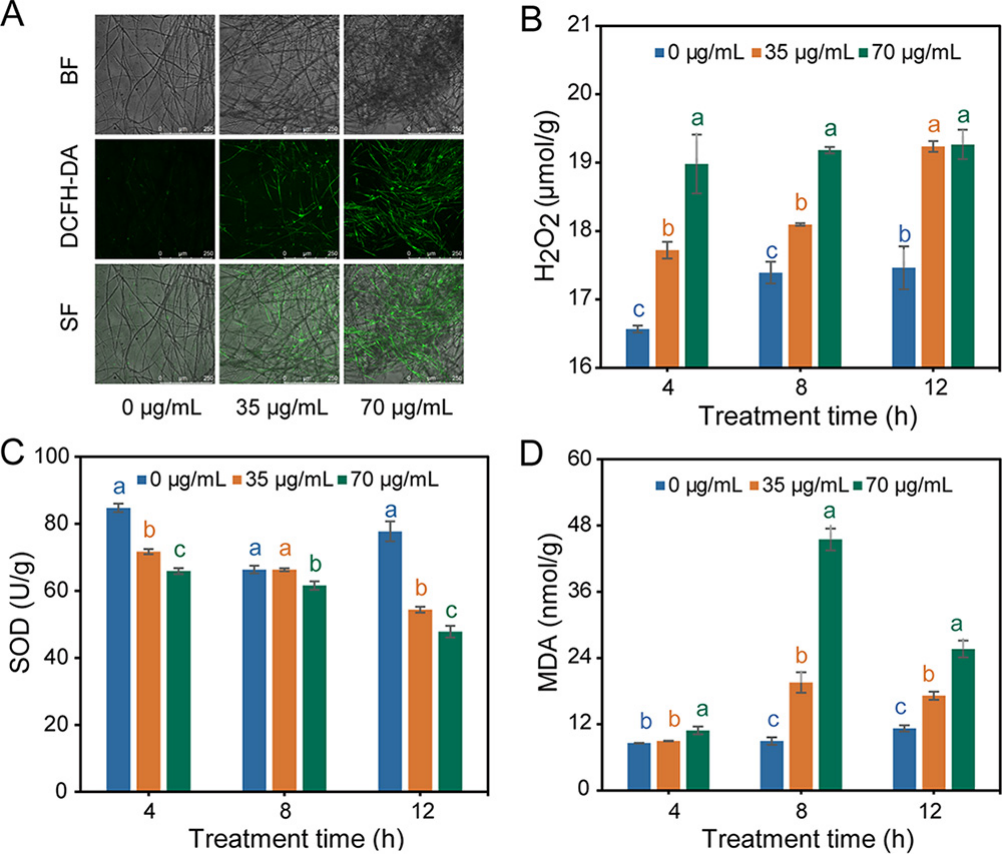
Effects of DDA (EC_50_ and 2-fold EC_50_) on the oxidative stress of *P. nicotianae*. (A) 2′,7′-DCFH-DA fluorescence staining. Magnification, ×100. BF, bright field; SF, superimposed field. (B) Hydrogen peroxide (H_2_O_2_) content. (C) Superoxide dismutase (SOD) enzyme activity. (D) MDA content. Values with different letters are statistically significantly different (*P* < 0.05).

ROS level and antioxidant enzyme activities were measured to evaluate the effect of DDA on oxidative stress of *P. nicotianae*. Using 2′,7′-dichlorodihydrofluorescein diacetate (DCFH-DA), a fluorescent dye widely used to detect endogenous ROS production, we showed that the fluorescence density of mycelia increased notably upon DDA treatment, whereas only a small amount of fluorescence was observed in the control group (Fig. 5A). In addition, the content of hydrogen peroxide (H_2_O_2_), an important ROS, in mycelia of the treatment group greatly exceeded that of the control group in a dose-dependent manner (Fig. 5B). In turn, the effect of the DDA on antioxidant enzyme (SOD) activity of *P. nicotianae* is shown in Fig. 5C. The SOD activity of the treatment group was consistently lower than that of the control and was negatively correlated with the DDA concentration (Fig. 5C). These results indicated that DDA-induced stress can inhibit SOD enzymatic activity, possibly inducing ROS accumulation in *P. nicotianae*. Moreover, the content of malondialdehyde (MDA), the end product of membrane lipid peroxidation, increased following treatment with DDA and was positively correlated with DDA concentration, indicating that the ROS accumulation induced by DDA resulted in cell membrane lipid peroxidation (Fig. 5D).

### Effects of DDA on cell membrane permeability of *P. nicotianae*.

**FIG 6:**
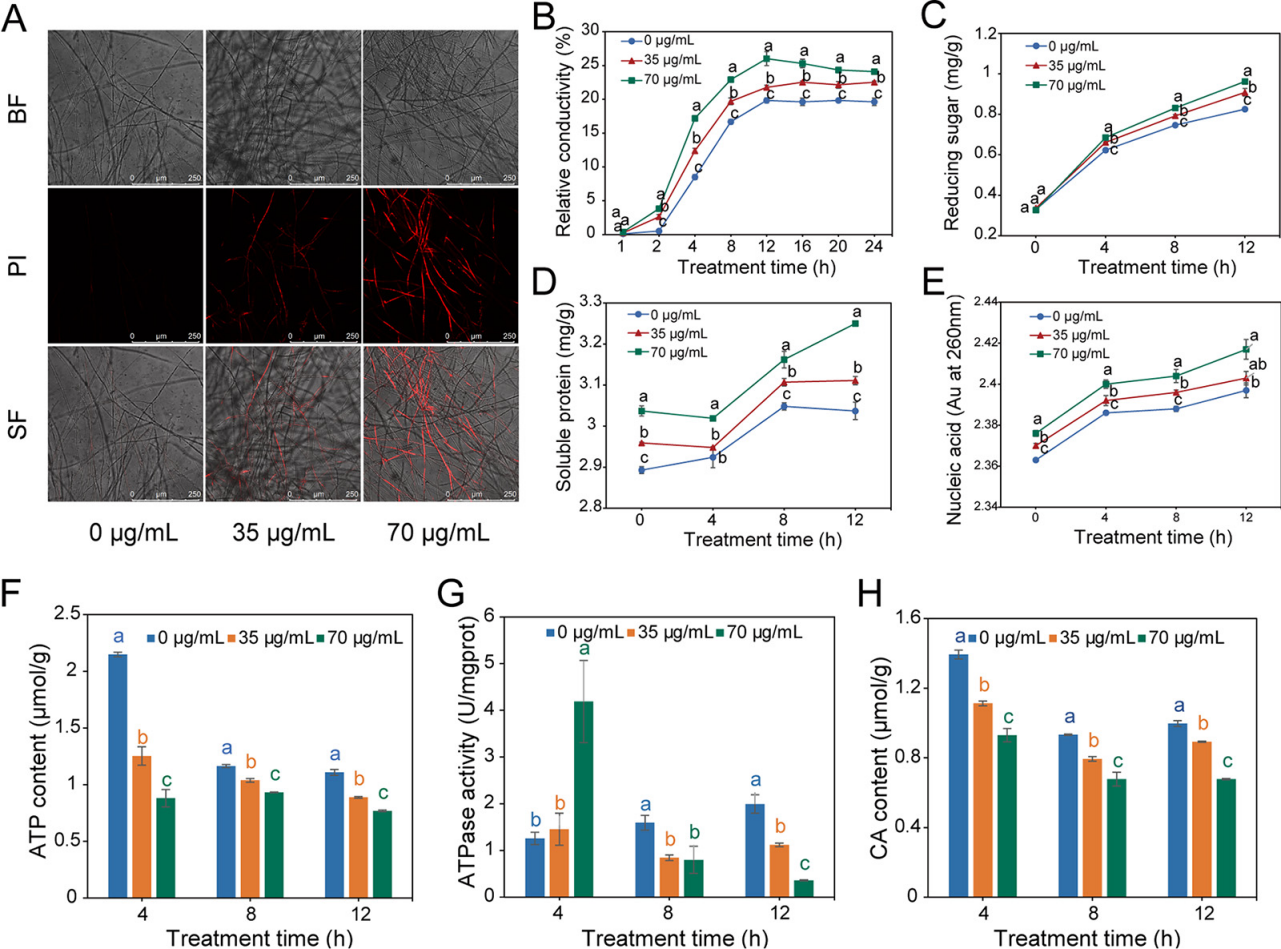
Effect of DDA treatment (EC_50_ and 2-fold EC_50_) on the cell membrane and energy metabolism of *P. nicotianae*. (A) Propidium iodide (PI) staining. Magnification, ×100. BF, bright field; SF, superimposed field. (B) Relative conductivity. (C) Reducing sugar. (D) Soluble protein. (E) Nucleic acid content. (F) ATP content. (G) ATPase activity. (H) Citrate acid (CA) content. Values with different letters are statistically significantly different (*P* < 0.05).

To observe the changes in cell membrane permeability, propidium iodide (PI) staining was performed. Strong fluorescence was detected in the mycelia exposed to DDA for 12 h and was enhanced with increasing DDA concentration, whereas minimal fluorescence was detected in the control group (Fig. 6A). The results indicated that DDA decreased mycelial viability and destroyed cell membrane integrity. Furthermore, the effect of DDA on the cell membrane permeability of *P. nicotianae* was verified by detecting the relative conductivity and cellular contents in the supernatant (Fig. 6B). With the increase in DDA concentration and extension of treatment time, the relative conductivity and the cellular contents (reducing sugars, soluble proteins, and nucleic acids) of the treated group were significantly higher than those of the control group (Fig. 6C to E). Therefore, we speculated that DDA could destroy cell membrane permeability and cause cell content leakage, leading to an increase in relative extracellular conductivity.

### Effects of DDA on ATP content, ATPase activity, and CA content.

The ATP content, ATPase activity, and citric acid (CA) content were employed to examine the effect of DDA on energy metabolism. The changing trends of ATP in the DDA treatment and control groups were comparable, with ATP content decreasing gradually with the increase in DDA concentration and extension of processing time (Fig. 6F). Nevertheless, the ATPase activity in the treated groups first increased and subsequently decreased, which differed markedly from the increasing trend of the control. One possibility to explain this phenomenon could be that the ATPase activity first increased due to the DDA-induced stress and then decreased because of the seriously damaged energy system (Fig. 6G). In addition, the content of CA, an important intermediate in the TCA cycle, was reduced by DDA in a concentration-dependent manner (Fig. 6H). These results revealed that DDA might inhibit the growth of *P. nicotianae* by disturbing energy metabolism.

## DISCUSSION

The role of MCFAs in resisting plant pathogens has long been underestimated. To date, few MCFAs and their derivatives have been developed as plant disease protection agents. Previous studies have reported that some MCFAs, including heptanoic, octanoic, nonanoic, and lauric acids, have antifungal activities against phytopathogens (14–17). In the present study, DDA significantly inhibited the growth of *P. nicotianae* and *P. capsici in vitro*, whereas it had relatively weak effects on other common plant-pathogenic fungi, indicating that DDA exerted specific antimicrobial activity against oomycetes of *Phytophthora* spp. To the best of our knowledge, this represents the first report of the inhibitory activity of DDA against plant pathogens. Our results revealed for the first time that DDA could exhibit significantly specific inhibitory activity against oomycetes, far better than that of other MCFAs reported previously (16), thereby highlighting DDA as a potential selective agent against oomycete pathogens.

*In vivo* analysis demonstrated that with the addition of DDA, the incidence of tobacco black shank disease decreased significantly (77.06% with an 800-fold DDA dilution). Moreover, the DNA copy numbers of *P. nicotianae* in the rhizosphere soil of tobacco seedlings decreased markedly, which suggested that DDA inhibited the colonization of *P. nicotianae* in the root, protecting tobacco from pathogen infection. The results showed that reducing the number of pathogens in the soil to improve the control effect of soilborne diseases may constitute an effective mechanism of biological control, which was consistent with previous research results. Sa et al. (18) found that the number of Fusarium oxysporum organisms in rhizosphere soil treated with B. subtilis N6-34 decreased significantly compared with that in the blank control and showed a gradual decrease in the number of F. oxysporum organisms with increasing inoculation time. In addition, based on high-throughput sequencing technology, it was found that the abundance of Oomycota in rhizosphere soil markedly decreased following DDA and METM treatment. Moreover, the relative abundances of *Phytophthora* and *Globisporangium*, oomycete pathogens of many economically important vegetable crops (19–21), were also significantly decreased in DDA-treated groups compared with control and METM groups, which could support the hypothesis that DDA acts as a specific oomycete inhibitor.

It has been demonstrated that the primary antimicrobial mode of MCFAs is cell membrane disruption consequent to its amphipathic nature (4, 22, 23). Previous studies have suggested that some long-chain fatty acids can influence enzyme activities (22), fatty acid metabolism (24), and virulence gene expression (25). Bhattacharyya et al. (4) found that caprylic acid and its derivative could penetrate and disturb the fungal membrane, thereby inhibiting fungal growth. Alsammarraie et al. (12) verified that deformation of cell structures, such as cell wall and plasma membrane, kills microorganisms. In the present study, SEM images revealed that the mycelia of the control group were homogeneous and smooth, with regular and intact cell walls and membranes. In contrast, DDA treatment led to distortion, irregular shrinkage, visible collapse, and damage to the mycelial surface. TEM images showed that DDA caused cell deformation, cell wall and membrane blurring, and organelle damage. Tannic acid dyeing results confirmed that DDA could disrupt the integrity of the mycelial cell wall. However, the underlying mechanisms of MCFAs are poorly understood. This study revealed multiple modes of action of DDA against *P. nicotianae*, including oxidative stress, membrane homeostasis, and energy and substance metabolism, which provides a new perspective for developing fatty acid biopesticides against plant oomycete pathogens.

Oxidative stress may play a critical part within in the inhibitory effects of DDA against *P. nicotianae*. Antimicrobial mechanisms induced by ROS have been previously observed (13, 26, 27). Cai et al. (3) found that g-C_3_N_4_ nanosheets generated a large amount of ROS and induced the formation of intracellular ROS, leading to oxidative stress, autophagy, slow metabolism, and growth suppression in *P. capsici*. ROS are intracellular metabolism products, playing a vital part as secondary messengers in many signal pathways. However, excessive ROS can cause enzyme inactivation and membrane disruption, promote apoptosis, and even lead to cell death (28–30). The degree of biological damage caused by oxidation is reflected by the cellular content of MDA, the final product of lipid peroxidation (31). As predicted, DCFH-DA fluorescence staining, H_2_O_2_ measurement, and MDA content determination showed that cells exposed to DDA demonstrated a notable increase in the levels of ROS and MDA, suggesting the presence of induced oxidative damage (i.e., lipid peroxidation) in *P. nicotianae*. Furthermore, genes associated with antioxidant enzymes, including SOD, cytochrome *c* peroxidase, and catalase (CAT), were significantly downregulated, indicating that antioxidant defenses based on antioxidant enzymes were compromised, which was confirmed by the determination of SOD enzyme activity. These results suggested that DDA-induced stress inhibited oxidoreductase activity and provoked a large amount of ROS accumulation in mycelia, resulting in an imbalance of ROS metabolic processes in *P. nicotianae*. In turn, oxidation injury caused by the accumulation of large amounts of ROS reduces mycelial growth rate, destroys cell integrity, and diminishes reactive oxygen degradation (32–34).

Mitochondria are a key source of intracellular ROS; however, damaged mitochondria produce excessive ROS, which could cause oxidative stress, energy suppression, and cell death. The increase in ROS levels in *P. nicotianae* indicated the potential damage to mitochondria caused by DDA. Succinic acid, a molecule that inhibits ROS-mediated lipid peroxidation in mitochondria (35), was decreased by DDA treatment. Similarly, significant transcription of *MPV17* (mediating antioxidant and antiapoptotic functions in mitochondria) was also observed. In addition, the inhibition of energy metabolism as revealed through our transcriptome and metabolome analyses further demonstrated the potential harmfulness of DDA to mitochondria, as these organelles represent vital energy-producing centers in eukaryotes to provide energy for cells (36–38). Consistent with this, we found that the expression of key enzyme-coding genes in the energy metabolism pathways was dramatically downregulated upon DDA treatment, including pyruvate metabolism, glyoxylate and dicarboxylate metabolism, the TCA cycle, and sulfur metabolism. Moreover, the key metabolites related to these processes, such as succinic, oxalic, and citric acids, decreased significantly. This observation was confirmed by the downregulated ATP content, ATPase activity, and CA content. Similar to our findings, microbial metabolites have previously been reported to play an antimicrobial role by destroying the energy metabolism of pathogens. Jiang et al. (39) demonstrated that iturin A from Bacillus subtilis led to the swelling of Aspergillus carbonarius mitochondria, which may reduce energy production and trigger apoptosis. In general, these results suggested that DDA led to mitochondrial dysfunction in *P. nicotianae*, resulting in reduced ATP synthesis, increased oxidative stress, and apoptosis (40–43).

Our findings indicated that the cellular membrane may be one of the targets of DDA, as DDA-induced stress disrupted the physiological metabolism of membrane components. Previous studies have reported that fatty acids could act as inhibitors of ergosterol (the main component in fungal membranes) biosynthesis (44, 45). In contrast to fungi, the cell membrane of oomycetes is mainly composed of phospholipids rather than ergosterol. Following DDA treatment, genes related to the membrane component synthesis of *P. nicotianae* (*PCYT1* and *PTDSS2*) were significantly downregulated, such as phosphorylcholine cytidylyltransferase (CTP) in glycerophospholipid metabolism (map00564), which affects the synthesis of phosphatidylcholine (PC). PC is an important lipid active component in phospholipids, with CTP serving as the rate-limiting enzyme for PC synthesis (46, 47). The neutral ceramidase gene in sphingolipid metabolism (map00600, *ASAH2*) was also downregulated, indicating that synthesis of sphingolipid, another important component of the cell membrane, may be blocked (48). Moreover, phospholipase gene expression (*TGL4*) was upregulated, which may result in the self-degradation of phospholipids and the accumulation of phospholipid degradation products (49) such as malonic, myristic, and palmitoleic acids. In addition, the cytoskeleton has a large role in sustaining the cell structure. Genes encoding tubulin (*TUBA*) were significantly downregulated upon DDA treatment, indicating that DDA inhibits the normal synthesis of the cytoskeleton, leading to increased instability and vulnerability of cell membranes to disruption (50). Furthermore, the cell membrane undertakes the function of transporting various substances across the membrane (51). Previous studies have shown that altered membrane permeability could reduce the transmembrane potential, inhibit the transmembrane transport proteins (ATPase and ABC transporter proteins), and affect the signaling pathway (MAPK signaling pathway), resulting in the dysfunction of normal cells (52). Together, these results demonstrate that the interference in membrane lipid metabolism mediated by DDA destroyed the membrane integrity of *P. nicotianae*. Moreover, the enhancement of PI staining fluorescence intensity, the increase of relative conductivity of the mycelial supernatant, and the leakage of cell contents in the DDA group corroborated the assumption that the cell membrane structure and integrity were disrupted. Overall, the destruction of membrane homeostasis may constitute an important mechanism by which DDA inhibits *P. nicotianae*.

**FIG 7:**
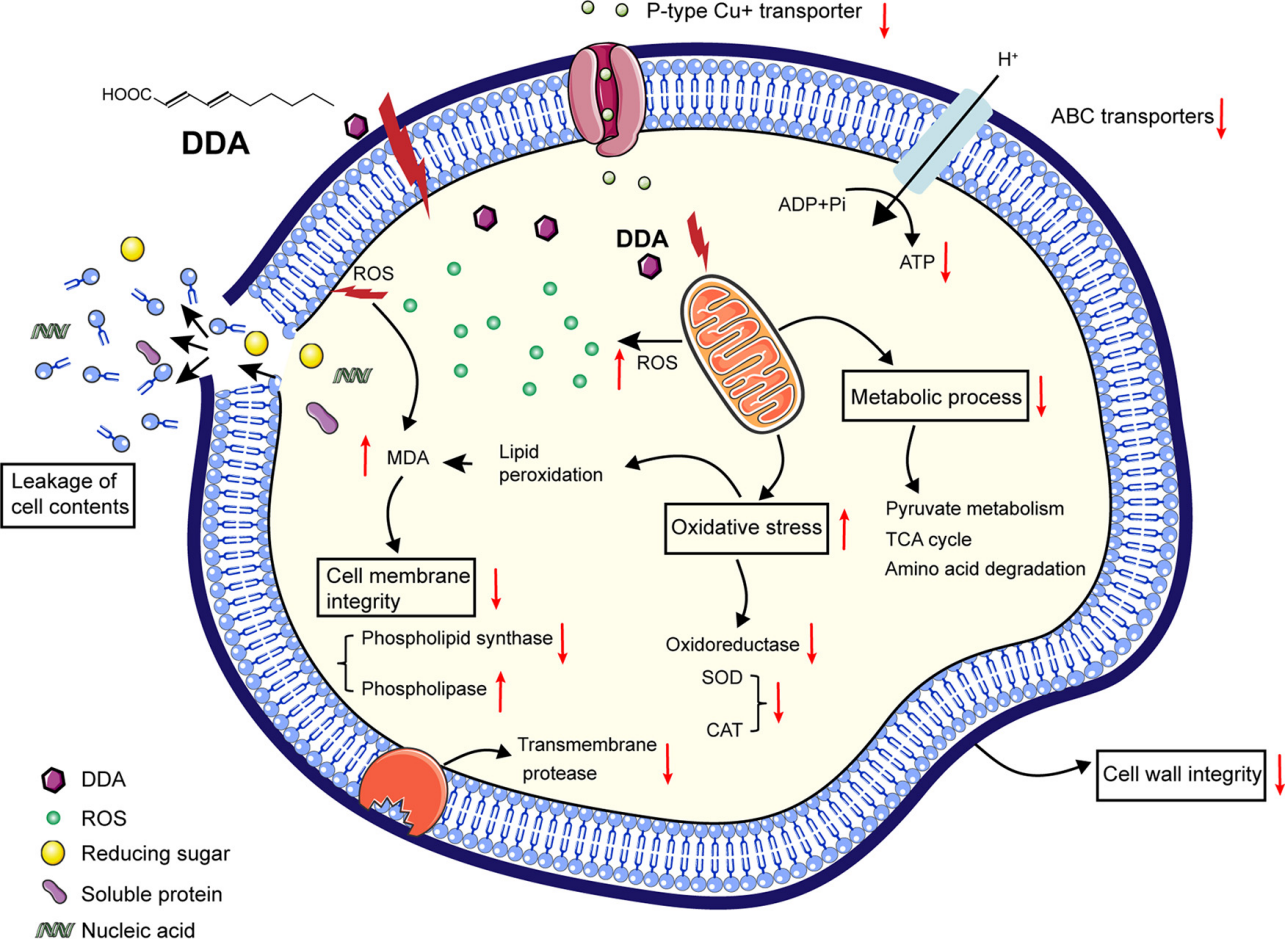
Model diagram of DDA acting on *P. nicotianae*. The cell membrane and mitochondria are attacked by DDA, resulting in ROS accumulation, leading to cell membrane peroxidation, reduced activity of transporters on the membrane, aggravated cell membrane damage, and leakage of cell contents, thereby interfering with cell energy utilization and metabolic pathways.

In summary, we isolated an MCFA, DDA, from the coculture fermentation liquor of *T. asperellum* HG1 and B. subtilis Tpb55, which exhibits effective and specific antimicrobial activity against *P. nicotianae*. Moreover, *in vivo* analysis demonstrated that DDA could significantly reduce the occurrence of tobacco black shank caused by *P. nicotianae* and reduce the abundance of oomycetes in rhizosphere soil. In addition, the results of transcription, metabolism, and physiological indices of *P. nicotianae* treated with DDA verified that DDA may attack the cell membrane and mitochondria, possibly causing devastation of the cell membrane integrity and excessive accumulation of ROS, further aggravating cell membrane damage and interfering with cell energy utilization and metabolic pathways, leading to the inactivation or death of *P. nicotianae* (Fig. 7). Overall, this study highlighted DDA as a potential antimicrobial agent against *P. nicotianae* that also holds promise as a new oomycete inhibitor.

## MATERIALS AND METHODS

### Strains.

*P. nicotianae* JM01 (PRJNA389504) was isolated from tobacco infected with tobacco black shank, cultivated on oat agar (OA) medium at 28°C (53), and deposited at the Marine Agriculture Research Center, Tobacco Research Institute of the Chinese Academy of Agricultural Sciences, Qingdao, China. *T. asperellum* HG1 (CGMCC 19276) and B. subtilis Tpb55 (CGMCC 2843) were isolated from Hainan mangrove samples and the surface of tobacco leaves, respectively, and had been cultured on potato dextrose agar (PDA) medium and Luria-Bertani agar (LA) medium (Qingdao Hope Bio-Technology Co., Ltd., Qingdao, China) at 28°C for further study (54). *T. asperellum* HG1 and B. subtilis Tpb55 were deposited at the China General Microbiological Culture Collection Center (CGMCC), Beijing, China.

### Coculture conditions.

In this study, liquid nutrient broth (N8300; Solarbio Science & Technology Co., Ltd., Beijing, China) was utilized for coculturing. The spores of *T. asperellum* HG1 were obtained by washing the 5-day-old culture with sterile water, followed by filtration through degreasing cotton. The number of spores was counted using an automatic cell counter (Countstar IC1000; Countstar, Shanghai, China), and diluted to 1 × 10^6^ spores/mL as a spore suspension. B. subtilis Tpb55 was transferred from 2-day-old culture in LA medium into nutrient broth (NB) and incubated overnight (28°C, 180 rpm/min), following which the number of cells was measured using a microplate reader (SP-Max 2300A2; Flash, China), and cultures were diluted to 1 × 10^6^ CFU/mL as a seed liquid. The spore suspension of *T. asperellum* HG1 (5 mL, 10^6^/mL) was inoculated in NB medium (250 mL/500-mL bottle) and cultured for 24 h (28°C and 180 rpm), followed by inoculation with B. subtilis Tpb55 (0.5 mL, 10^6^/mL); the fermentation was continued for 7 days.

### Isolation and identification of DDA.

The fermentation filtrate from the coculture of *T. asperellum* HG1 and B. subtilis Tpb55 was extracted with ethyl acetate (EtOAc) three times and then evaporated under vacuum to obtain the final extract (9.4 g). The extract was separated using a Sephadex LH-20 chromatography column (CC) (CH_2_Cl_2_-methanol [MeOH], 1:1 [vol/vol]) after being eluted on a silica gel CC with 10% EtOAc-petroleum ether. It was further purified by HPLC (Waters e2695-2998; Waters, Milford, MA, USA) using MeOH-H_2_O (75%) with 0.1% trifluoroacetic acid (TFA) to yield DDA (3.2 mg). The DDA structure was identified using ^1^H and ^13^C NMR (DD2 500 MHz NMR spectrometer; Agilent Technologies, Santa Clara, CA, USA) and electrospray ionization (ESI) (Micromass Q-TOF spectrometer; Waters) spectra and compared to the literature and the standard compound purchased from Toronto Research Chemicals Inc. (North York, ON, Canada). ^1^H NMR (dimethyl sulfoxide-*d*_6_ [DMSO-*d*_6_], 500 MHz) data are as follows: *δ* 12.16 (s), 7.14 (1H, dd, *J *= 15.5, 10.0 Hz), 6.22 (1H, dd, *J *= 15.5, 10.0 Hz), 6.17 to 6.27 (1H, m), 5.78 (1H, d, *J *= 15.5 Hz), 2.13 (2H, q, *J *= 7.0 Hz), 1.39 (2H, dt, *J *= 14.5, 7.5 Hz), 1.23 to 1.30 (4H, m), 0.86 (3H, t, *J *= 7.0 Hz). ^13^C NMR (DMSO-*d*_6_, 125 MHz) data are as follows: *δ* 167.7 (C), 144.6 (CH), 144.2 (CH), 128.3 (CH), 120.1 (CH), 32.3 (CH_2_), 30.9 (CH_2_), 27.9 (CH_2_), 21.9 (CH_2_), 13.9 (CH_3_); ESIMS *m/z* 169.11 (M+H)^+^.

### Synthesis and purification of DDA.

Jones reagent (8.5 mL, 1.5 eq; J&K Scientific Ltd.) was added dropwise to a stirred solution of 2*E*,4*E*-deca-2,4-dienal (2 mL, 1.0 eq; J&K Scientific Ltd., Shanghai, China) with 25 mL acetone at 0°C. The reaction mixture was agitated at 0°C for 10 min before being moved to room temperature (25°C) for stirring overnight, followed by filtration to remove the residue and rinsing three times with 5 mL acetone. The filtrate was dried and redissolved in 20 mL EtOAc. The organic solution was washed three times with distilled water (10 mL); subsequently, 4 M NaOH was added to the EtOAc phase until a pH of 8 to 9 was reached and extracted with 10 mL distilled water (three times). Hydrochloric acid (4 M) was added to the combined aqueous phases until the pH reached 1 to 2. The solution was extracted with 10 mL EtOAc (three times), and the mixed organic extracts were washed with saturated NaCl solution (20 mL, three times) and then condensed in a vacuum to yield residues. The reaction was monitored by thin-layer chromatography (TLC) using Yantai Jiangyou (Yantai, China) GF254 silica gel plates. The residues obtained were initially purified by silica gel CC with gradient elution of EtOAc-petroleum ether from 10% to 100%, then separated on an ODS column eluted with 30 to 90% MeOH-H_2_O, and purified on Sephadex LH-20 CC (CH_2_Cl_2_/MeOH, 1/1[vol/vol]), followed by purification by HPLC using 70% MeOH-H_2_O plus 0.1% TFA to yield DDA. The instrument data were identical to those of the standard compound.

### Assay of activity against *P. nicotianae*.

The inhibitory activity of DDA against *P. nicotianae* was estimated using the mycelial growth rate method (55). Briefly, DDA dissolved in DMSO was added to OA medium (0.5% DMSO [vol/vol]) to a series of final concentrations (0, 12.5, 25, 50, 100, 200, and 400 μg/mL). Mycelial plugs (5-mm diameter) were cut from the edge of a 3-day-old colony, inoculated on the center of OA plates, and then incubated at 28°C for 3 days. Each treatment was replicated three times. The mycelial diameter was measured using the cross method (56) to calculate inhibition rate (57) and EC_50_ (effective concentration for 50% inhibition of mycelial growth). EC_50_ was obtained using the toxicity regression equation (58). The inhibition rate was calculated as [(*R* – *r*)/*R*] × 100, where *R* and *r* are the average diameters of the control and treatment colonies, respectively.

### Antimicrobial spectrum of DDA.

The inhibitory rate of DDA (100 μg/mL) against some pathogenic oomycetes (*P. capsici*, *P. sojae*, *P. aphanidermatum*, *P. coloratum*, and *P. ultimum*) and fungi (A. alternata, *B. cinerea*, F. graminearum, *C. cornigerum*, M. oryzae, and *P. piricola* Nose) were determined using the mycelial growth rate method to evaluate its antimicrobial activity. Among them, *P. capsici* and *P. sojae* were cultured on OA, and others were cultured on PDA. The dissolution method for DDA, strain culture conditions, and inhibition rate were the same as those for *P. nicotianae*.

### Mycelial ultrastructure observation.

SEM and TEM were implemented according to previously detailed methods (59–61). The DDA treatment method was the same as that described above to obtain media with final concentrations of 35 (EC_50_) and 70 (2× EC_50_) μg/mL. An equal volume of 0.5% DMSO served as a control. Then, the plug of *P. nicotianae* (5 mm) was inoculated and incubated at 28°C for 3 days.

For SEM, collected mycelia were fixed with 2.5% glutaraldehyde fixing solution (4°C, 4 h) and washed six times with 0.01 M phosphate buffer solution (PBS) for 20 min each, followed by dehydration with graded ethanol aqueous solutions (30% to 100%) for 30 min at each concentration. Subsequently, samples were transferred to isoamyl acetate, dried at the critical point of carbon dioxide, plated with gold, and then observed using a JSM-840 SEM (JEOL, Tokyo, Japan).

For TEM, samples were fixed with 2.5% glutaraldehyde and 1% osmic acid for 4 and 2 h, respectively, washed with 0.01 M PBS, and then dehydrated in ethanol with a gradient series for 30 min. Shortly thereafter, samples were embedded in Epon812 and polymerized, followed by ultrathin sectioning. After being dyed with uranyl acetate and lead nitrate, samples were observed using a JEM-1200EX TEM (JEOL).

### *In vivo* test.

A pot experiment was used to verify the control effect of DDA on tobacco black shank. The inoculum of the pathogen and the diseased soil were prepared according to the method described by Zhang et al. (62). Briefly, millet was sterilized after boiling for 30 min and inoculated with *P. nicotianae*, followed by incubation for 14 days at 25°C. Tobacco seedlings with 4 or 5 leaves were transplanted into the pots (one plant per pot) and immediately treated with 20 mL of the agent. Each pot contained a mixture of field soil (200 g) and millet with *P. nicotianae* (0.8 g). The experiment included four treatments: distilled water (C), 1,600-fold dilution of DDA (LDDA), 800-fold dilution of DDA (HDDA) and 800-fold dilution of metalaxyl-M (METM). Every agent was dissolved with 0.5% DMSO and diluted with water. Each treatment was repeated three times for 15 tobacco seedlings. Subsequently, the seedlings were cultivated in a greenhouse (28°C, 70% relative humidity). On the 7th day after pathogen inoculation, the incidence of tobacco black shank was evaluated, and the DNA copy numbers of *P. nicotianae* and high-throughput sequencing of oomycetes were obtained from rhizosphere soil.

### Incidence survey on tobacco black shank.

According to Zhang et al. (53), the severity of tobacco black shank is divided into different grades, where 0 indicates no symptoms, 1 indicates that less than one-third of the total leaves are wilted, 3 indicates that one-third to one-half of the total leaves are wilted, 5 indicates that one-half to two-thirds of the total leaves are wilted, 7 indicates that more than two-thirds of total leaves are wilted, and 9 indicates that the plant was dead. The incidence of disease with different treatments was investigated, and the disease index was analyzed as follows: disease index = [Σ(disease grade × number of diseased plants at each grade)]/(total number of plants in each treatment × 9).

### Real-time qPCR of *P. nicotianae*.

On the 7th day after potting, the DNA copy numbers of *P. nicotianae* in rhizosphere soil were determined by qPCR in three biological and three technical replicates. The primers PNF (5′-TGAAGAACGCTGCGAACTGC-3′) and PNR (5′-CTGACATCTCCTCCACCGACTA-3′) were used to amplify a 172-bp specific fragment of *P. nicotianae*. DNA was extracted and purified using the DNeasy PowerSoil kit (Qiagen, Hilden, Germany). Amplifications were performed in a total volume of 20 μL containing 10.0 μL SYBR premix (TaKaRa, Shiga, Japan), 0.4 μL ROX reference dye (50×), 2.0 μL DNA, 0.4 μL of each primer (10 μM/μL), and 6.8 μL double-distilled water (ddH_2_O) on the ABI-7500 real-time PCR system (Thermo Fisher, Waltham, MA, USA). The amplification procedure was as follows: 94°C for 5 min, 94°C for 20 s, 65°C for 40 s, and 72°C for 40 s, with a total of 40 cycles. Plasmids containing the specific fragment were constructed and diluted to different concentrations to prepare the standard curve, and the DNA copy numbers of *P. nicotianae* of each sample were calculated according to the standard curve equation and the cycle threshold (*C_T_*) values of the samples.

### DNA extraction, PCR amplification, and high-throughput sequencing of oomycetes.

The rhizosphere soil samples were collected as reported by Zheng et al. (63). All samples were stored at −80°C until DNA extraction. A total of 12 samples were collected, including three treatments (C, HDDA, and METM) and three replicates. DNA from the samples was extracted using the FastDNA spin kit (MP Biomedicals, Irvine, CA, USA) according to the manufacturer’s instructions. The concentration and integrity of DNA were evaluated using a NanoDrop 2000 (Thermo Scientific, Wilmington, DE, USA) and agarose gel electrophoresis, respectively. Primers ITS1OF (5′-CGGAAGGATCATTACCAC-3′) and SORevR (5′-AGCCTAGACATCCACTGCTG-3′) targeting the oomycetal *ITS1* region were used for amplicon sequencing (64). The PCR system (20 mL) contained 5×FastPfu buffer (4 μL), 2.5 mM concentrations of deoxynucleoside triphosphates (dNTPs) (2 μL), a 5 μM concentration of each primer (0.8 μL), FastPfu polymerase (0.4 μL), bovine serum albumin (BSA) (0.2 mL), and template DNA (10 ng). PCR was performed in triplicate at 95°C for 3 min, followed by 35 cycles of 95°C for 30 s, 55°C for 30 s, and 72°C for 45 s and a final extension step of 72°C for 10 min. PCR products were purified, and sequenced on the Illumina NovaSeq PE250 platform at the Majorbio Bio-Pharm Technology Co., Ltd. (Shanghai, China).

For the sequencing data obtained, fastp (v0.20.0) was used for quality control; sequence splicing was performed using Flash (v1.2.7) software, and the sequences were clustered into OTUs (97% similarity) using UPARSE software (v7.1). Each sequence was classified at the species level using the RDP classifier (v2.11) and annotated against the NCBI_nt database (20210917) with QIIME (v1.91). R (v3.3.1) was used for community composition analysis, PCoA, and intergroup flora difference analysis.

### RNA-Seq analysis.

*P. nicotianae* was preincubated on OA for 4 days, and the harvested mycelia (1.5 g) were treated with 35 μg/mL of DDA in oat liquid medium (30 mL) for 24 h. This experiment was conducted with three biological replicates. Collected mycelia were washed three times with PBS and frozen with liquid nitrogen for RNA-Seq analysis. Mycelia without DDA treatment served as the control. Total RNA was extracted from tissue using the TRIzol reagent. After RNA quality was determined using the NanoDrop 2000 and 2100 Bioanalyzer (Agilent), the RNA-Seq library was constructed using the TruSeq RNA sample preparation kit from Illumina (San Diego, CA, USA), followed by sequencing on the Illumina NovaSeq 6000 sequencer (2 × 150 bp).

Low-quality reads were removed, and the clean reads were used for subsequent analysis, which were mapped to the *P. nicotianae* reference genome (GenBank assembly accession: GCA_001482985.1). RNA-seq by expectation-maximization (RSEM) was used to obtain the read counts of each gene, and the number of transcripts per million reads (TPM) was used as an index to gauge expression (65). Based on the quantitative results of expression, the DEGs between the two groups were identify using the difference analysis software DEseq2, as well as the screening threshold | log_2_FC | ≥1 (FC is fold change) and adjusted *P* < 0.05 (66). The databases used to annotate DEGs were clusters of orthologous groups of proteins (COG), GO, and KEGG. GO enrichment and KEGG enrichment of DEGs were analyzed using Goatools and R. In addition, the Majorbio Cloud platform was utilized to analyze data. Additional details regarding the RNA-Seq analysis are provided in the supplemental material.

### qRT-PCR verification.

To validate the results of RNA-Seq, 10 genes were chosen for qRT-PCR verification (67). The sample preparation method was the same as that used for RNA-Seq. The extraction of total RNA of collected mycelia was the same as for RNA-Seq, and reverse transcription of each RNA sample was performed to obtain cDNA using the Evo M-MLV Mix kit with gDNA Clean for qPCR (Accurate Biology, Changsha, China). The qRT-PCR experiment was carried out using the TB Green Premix Ex Taq kit (TaKaRa) with the ABI-7500 real-time PCR system (Thermo Fisher). Primers are listed in Table S11. The β-actin gene was used as a stable reference gene to allow the relative quantification of the target gene (68); the expression levels of target genes were calculated using the 2^−ΔΔ^*^CT^* method.

### Metabolomic analysis.

The sample pretreatment and collection methods were as described for RNA-Seq analysis. Mycelia without DDA treatment were used as controls. The extraction method of metabolites was slightly modified, as described by Jin et al. (69). This experiment was performed with six biological replicates. A total of 50 mg mycelia was mixed with a 0.5-mL methanol-water solution (MeOH-H_2_O [vol/vol], 4:1, containing l-2-chloro-phenylalanine internal standard at 0.02 mg/mL), followed by grinding in a −20°C grinder with chloroform (50 Hz, 3 min). Samples were then extracted using an ultrasonic water bath and centrifuged (relative centrifugal force [RCF] of 13,000 at 4°C for 15 min). The supernatants were transferred into glass derivatization bottles and dried with nitrogen. A total of 80 μL methoxyamine pyridine hydrochloride solution (15 mg/mL) was added to the bottles, which were vortexed for 2 min, followed by oximation in a shaking incubator (37°C, 90 min). Subsequently, the samples were mixed with 80 μL of the derivatization reagent bis(trimethylsilyl)trifluoroacetamide (BSTFA; containing 1% trimethylchlorosilane [TMCs]), and reacted at 70°C for 60 min after being vortexed for 2 min. Subsequently, the samples were placed at room temperature for 30 min and used for further analysis.

After derivatization, the samples were detected using GC-MS (8890B-5977B; Agilent). Original documents were preprocessed using the MassHunter workstation quantitative analysis software (v10.0.707.0). The sample relationships were analyzed by OPLS-DA. Student's *t* test (unpaired) analysis was performed on metabolites, and metabolites with VIP values of >1 and *P* values of <0.05 were considered DEMs (70, 71). The data were further analyzed using the Majorbio Cloud platform. Additional details regarding the GC-MS analysis are provided in the supplemental material.

### Sample preparation for physiological index detection.

*P. nicotianae* was preincubated on OA for 4 days, and the harvested mycelia (1.5 g) were treated with 35 and 70 μg/mL of DDA in 0.01 M PBS (pH 7.4, 30 mL) for 4, 8, and 12 h (28°C, 180 rpm/min). Mycelia with 0.5% DMSO (vol/vol) served as the control. Each experiment was replicated three times. The supernatant and treated mycelia were used for subsequent physiological index detection.

### Effects of DDA on the cell wall integrity of *P. nicotianae*.

With slight adjustments, the integrity of the mycelial cell wall was assessed using the previously described tannic acid staining method (11). The treated mycelia were placed on a slide and dried at room temperature naturally. After 1 h of treatment with a 10% tannic acid solution, the samples were rinsed with distilled water. Subsequently, the samples were exposed to 5% crystal violet solution for 5 min before being washed with distilled water and stained with 5% Congo red solution for 2 min. Samples were allowed to dry at room temperature after rinsing, and the smear was prepared and observed using an optical microscope.

### Effects of DDA on the cytomembrane of *P. nicotianae*. (i) Observation of PI staining.

The integrity of the cell membrane was observed using PI staining (72). The treated mycelia were stained with PI (40 μg/mL) in the dark for 20 min, transferred to microscope slides, observed at 488 nm (excitation wavelength) and 617 nm (emission wavelength) using a laser scanning confocal microscope (TCS SP8 STED; Leica Microsystems, Wetzlar, Germany), and photographed.

### (ii) Assay of cell membrane permeability.

The relative conductivity of the mycelial supernatant treated with DDA was tested using a conductivity meter (DDJ-308A, INESA, Shanghai, China) to determine the permeability rate of the *P. nicotianae* cell membrane (73). The conductivity was measured immediately upon DDA addition to PBS containing mycelia and recorded as L0, and values at 1 to 24 h (28°C, 180 rpm/min) were recorded as L1. Moreover, the final conductivity after the mycelia were boiled for 30 min was tested, designated L2. The percent relative conductivity was calculated as [(L1 – L0)/(L2 – L0)] × 100.

### (iii) Assay of cellular leakage.

The cellular leakage of *P. nicotianae* was determined using a previously published method (74). The contents of reducing sugar (DNS method, 540 nm), soluble protein (Coomassie brilliant blue G-250, 595 nm), and nucleic acid (260 nm) in the treated mycelial supernatant were determined using a microplate reader (SP-Max 2300A2; Flash, Shanghai, China).

### Effects of DDA on oxidative stress of *P. nicotianae*.

DCFH-DA fluorescence staining was used to detect the level of ROS in mycelia. The treated mycelia were incubated in the dark for 30 min with DCFH-DA (final concentration, 10 μM). After washing with PBS buffer, the mycelia were observed using laser confocal microscopy (488 nm [excitation wavelength] and 525 nm [emission wavelength]) to measure ROS levels (72). Assay kits (BC3595, BC0175, BC0205, and BC0025; Solarbio Science & Technology Co., Ltd.) were utilized to measure H_2_O_2_ content, SOD activity, and MDA content, respectively, in treated mycelia.

### Effects of DDA on the energy metabolism of *P. nicotianae*.

ATP content, ATPase activity, and CA content were determined to verify the effects of DDA on the energy metabolism of *P. nicotianae*. The ATP and CA contents of treated mycelia were measured using assay kits (BC0305 and BC2155; Solarbio Science & Technology Co., Ltd.). ATPase activity was measured using the ATPase activity assay kit (A070-1-2; Nanjing Jiancheng Bioengineering Institute, Nanjing, China).

### Statistical analysis.

The mean values of all data were derived from three biological replicates, and the standard errors of the means were calculated. The significant differences between different treatments were analyzed by one-way analysis of variance (ANOVA) with Duncan’s test (*P* < 0.05) using SPSS 18.0 software. Student's *t* test was used to identify genera that showed significant differences in abundance between groups in the microbiome analysis. Tables and figures were processed using Microsoft Excel 2019, GraphPad Prism 9, and Adobe Illustrator 2020.

### Data availability.

Sequence data for RNA-Seq and high-throughput analysis in this study have been deposited in the NCBI Sequence Read Archive under BioProject numbers PRJNA812696 and PRJNA831376. The raw metabolic data have been deposited in the MetaboLights database under accession number MTBLS5405.
