# Supplementary material for: 2E,4E-Decadienoic Acid, a Novel Anti-Oomycete Agent from Coculture of Bacillus subtilis and Trichoderma asperellum
Source: Microbiol Spectr. 2022 Aug 9;10(4):e01542-22. doi: 10.1128/spectrum.01542-22 (PMC9430527; doi:10.1128/spectrum.01542-22)
Supplement: Supplemental file 1 — Supplemental material. Download spectrum.01542-22-s0001.pdf, PDF file, 0.9 MB [file spectrum.01542-22-s0001.pdf]

2*E*,4*E*-decadienoic acid, a novel anti-oomycete agent from co-culture of *Bacillus subtilis* and *Trichoderma asperellum*

Xi-Fen Zhang[#], Qing-Yu Li[#], Mei Wang, Si-Qi Ma, Yan-Fen Zheng, Yi-Qiang Li, Dong-Lin Zhao[*], Cheng-Sheng Zhang[*]

Tobacco Research Institute of Chinese Academy of Agricultural Sciences, Qingdao 266101, People's Republic of China

[#] Xi-Fen Zhang and Qing-Yu Li contributed equally to this work. Author order was determined on the basis of their contributions to this paper.

* To whom correspondence should be addressed. Tel: 86-532-88702115. E-mail: zhaodonglin@caas.cn (D.-L.Z.); zhangchengsheng@caas.cn (C.-S.Z.)

Running title: 2*E*,4*E*-decadienoic acid as a novel anti-oomycete agent

Captions

RNA-Seq Analysis.

Metabolite Analysis.

Fig. S1 ^{1}H NMR (500 MHz, DMSO-d_6) spectrum of DDA.

Fig. S2 ^{13}C NMR (125 MHz, DMSO-d_6) spectrum of DDA.

Fig. S3 ESIMS spectrum of DDA.

Fig. S4 Toxicity regression equation of DDA on *P. nicotianae*.

Fig. S5 Classification of DEGs.

Fig. S6 Validation of RNA-Seq results using qRT-PCR.

Table S1 Inhibition rates of Fr.1-7 (0.5 mg/mL) isolated from co-culture fermentation broth on *P. nicotianae*.

Table S2 Summary of RNA-seq reads in control (C) and treatment (T) groups of *P. nicotianae*.

Table S3 DEGs in GO enrichment analysis.

Table S4 DEGs in KEGG enrichment analysis.

Table S5 Summary of major genes and metabolites related to oxidative stress affected in *P. nicotianae* exposed to DDA compared to untreated groups.

Table S6 Summary of major genes and metabolites related to membrane homeostasis affected in *P. nicotianae* exposed to DDA compared to untreated groups.

Table S7 Summary of major genes and metabolites related to energy and substance metabolism affected in *P. nicotianae* exposed to DDA compared to untreated groups.

Table S8 Metabolite information detected by GC-MS. (Table shown in separate file)

Table S9 Information on the screening of differential metabolites.

Table S10 DEMs in KEGG enrichment analysis.

Table S11 Primers used for qRT-PCR analysis.

RNA-Seq Analysis. Total RNA was extracted from the tissue using TRIzol® Reagent (Plant RNA Purification Reagent for plant tissue) according the manufacturer's instructions (Invitrogen) and genomic DNA was removed using DNase I (TaKara). Then RNA quality was determined by 2100 Bioanalyser (Agilent) and quantified using the ND-2000 (NanoDrop Technologies). Only high-quality RNA sample (OD260/280=1.8~2.2, OD260/230≥2.0, RIN≥6.5, 28S:18S≥1.0, >1μg) was used to construct sequencing library.

RNA-seq transcriptome librariy was prepared following TruSeqTM RNA sample preparation Kit from Illumina (San Diego, CA) using 1μg of total RNA. Shortly, messenger RNA was isolated according to polyA selection method by oligo (dT) beads and then fragmented by fragmentation buffer firstly. Secondly double-stranded cDNA was synthesized using a SuperScript double-stranded cDNA synthesis kit (Invitrogen, CA) with random hexamer primers (Illumina). Then the synthesized cDNA was subjected to end-repair, phosphorylation and'A' base addition according to Illumina's library construction protocol. Libraries were size selected for cDNA target fragments of 300 bp on 2% Low Range Ultra Agarose followed by PCR amplified using Phusion DNA polymerase (NEB) for 15 PCR cycles. After quantified by TBS380, paired-end RNA-seq sequencing library was sequenced with the Illumina HiSeq xten/NovaSeq 6000 sequencer (2 × 150bp read length).

The raw paired end reads were trimmed and quality controlled by SeqPrep (https://github.com/jstjohn/SeqPrep) and Sickle (https://github.com/najoshi/sickle) with default parameters. Then clean reads were separately aligned to reference genome with orientation mode using HISAT2 (http://ccb.jhu.edu/software/hisat2/index.shtml) software. The mapped reads of each sample were assembled by StringTie(https://ccb.jhu.edu/software/stringtie/index.shtml?t=example) in a reference-based approach.

To identify DEGs (differential expression genes) between two different samples, the expression level of each transcript was calculated according to the transcripts per million reads (TPM) method. RSEM (http://deweylab.biostat.wisc.edu/rsem/) was used to quantify gene abundances. Essentially, differential expression analysis was performed using the DESeq2 with p-adjust ≤ 0.05, DEGs with |log2FC|>1 and p-adjust ≤ 0.05 (DESeq2) were considered to be significantly different expressed genes). In addition, functional-enrichment analysis including GO and KEGG were performed to identify which DEGs were significantly enriched in GO terms and metabolic pathways at Bonferroni-corrected P-value ≤0.05 compared with the whole-transcriptome background. GO functional

enrichment and KEGG pathway analysis were carried out by Goatools (https://github.com/tanghaibao/Goatools).

Metabolite Analysis. Fifty milligrams of samples were put into a 2 mL centrifuge tube and mixed with 0.5 mL methanol water solution (CH_3OH-H_2O v/v, 4:1, containing l-2-chloro-phenylalanine internal standard at 0.02 mg/mL). Then the samples were ground by steel ball in a –20°C grinder (50 Hz, 3 min). After that, ground samples were ground with 200 μL of chloroform (50 Hz, 3 min) and extracted by ultrasonic water bath for 30 min. After standing at 20°C for 30 min, the samples were centrifuged for 15 min (13000 RCF, 4°C). The supernatants were transferred into glass derivatization bottle and blown dry with nitrogen. Eighty microliters of methoxyamine pyridine hydrochloride solution (15 mg/mL) were added into bottles, vortexed for 2 min, and oxidated in a shaking incubator at 37°C for 90 min. The supernatants were taken out from incubator and mixed with 80 μL of BSTFA (containing 1% TMCs) derivatization reagent, vortexed for 2 min, and reacted at 70°C for 60 min. Afterwards, the samples were placed at room temperature for 30 min and used for GC/MS metabolomics analysis. The metabolome study used 6 replicates for the samples and 4 replicates for the blank samples (to remove impurities from the experiment).

Detection was performed using a gas chromatographer-mass spectrometer (GC-MS) (8890B-5977B, Agilent, USA). After derivatization, the samples were injected into the GC-MS system in split mode for analysis. The injection volume is 1 μL and the split ratio is 5:1. The sample was separated by passing through DB-5MS capillary column (40 m × 0.25 mm × 0.25 μm, Agilent 122-5532G) and detected by mass spectrometry. The temperature of the injection port is 260°C; the carrier gas is high-purity helium; the carrier gas flow rate is 1 mL/min; the septum purge flow rate is 3 mL/min; and the solvent delay is 5.5 min. Temperature rise procedure: the initial temperature was 60°C and maintained for 0.5 min, then the temperature was raised to 310°C at 8°C/min and maintained for 6 min. For the MS analysis, an electron bombardment ion source (EI) was used with a transmission line temperature of 310°C, an ion source temperature of 230°C, a quadrupole temperature of 150°C, an electron energy of 70 eV, and a scan range of 50-500 m/z in the full scan mode with a scan interval of 3.2 s.

The original documents of GC-MS were preprocessed by MassHunter workstation quantitative analysis (v10.0.707.0) software. Orthogonal partial least squares discriminant analysis (OPLS-DA) was used to analyze the sample relationships. Analysis of Student's t test (Unpaired) was performed on metabolites, and metabolites with VIP value > 1 and $P < 0.05$ were regarded as differentially

expressed metabolites (DEMs)

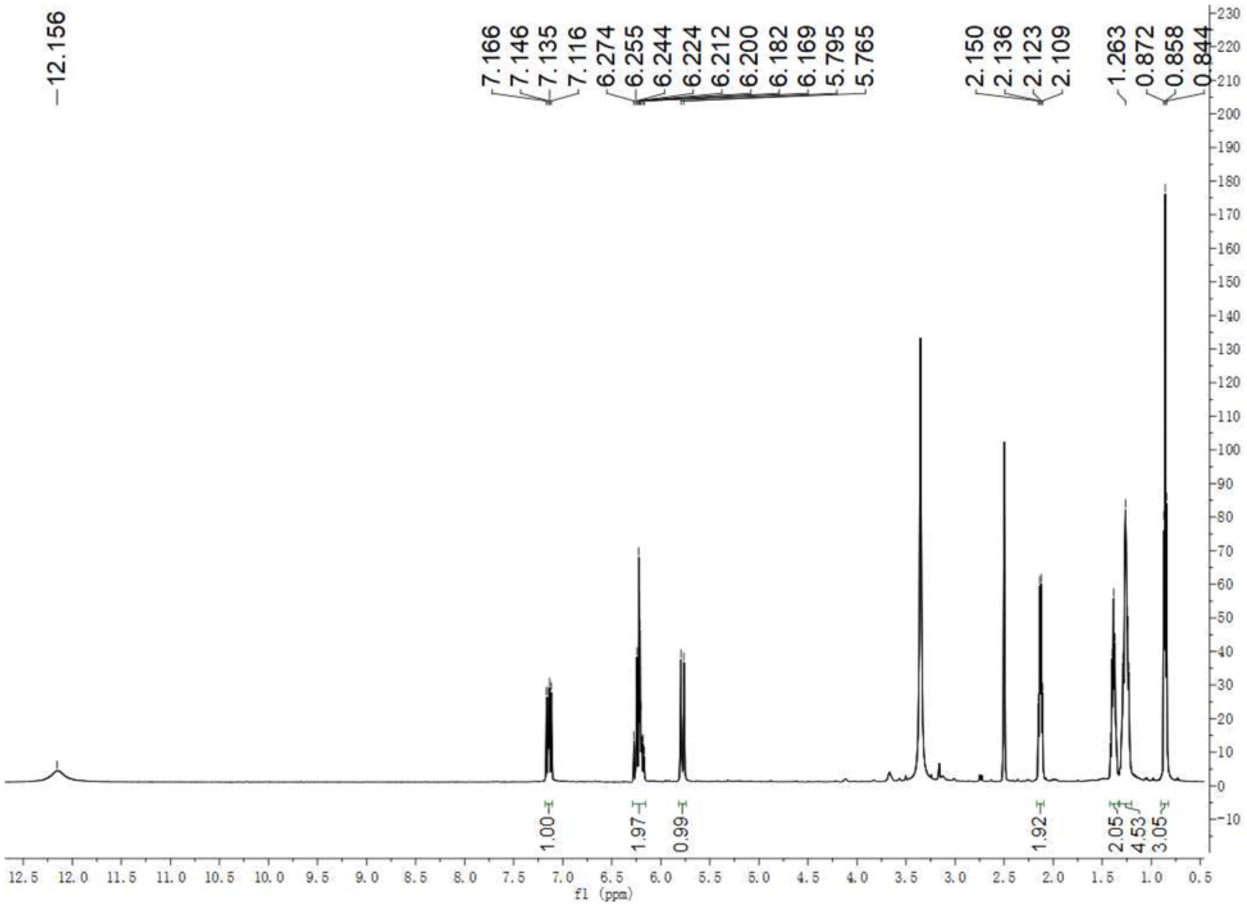

Fig. S1 ^{1}H NMR (500 MHz, DMSO-d_6) spectrum of DDA.

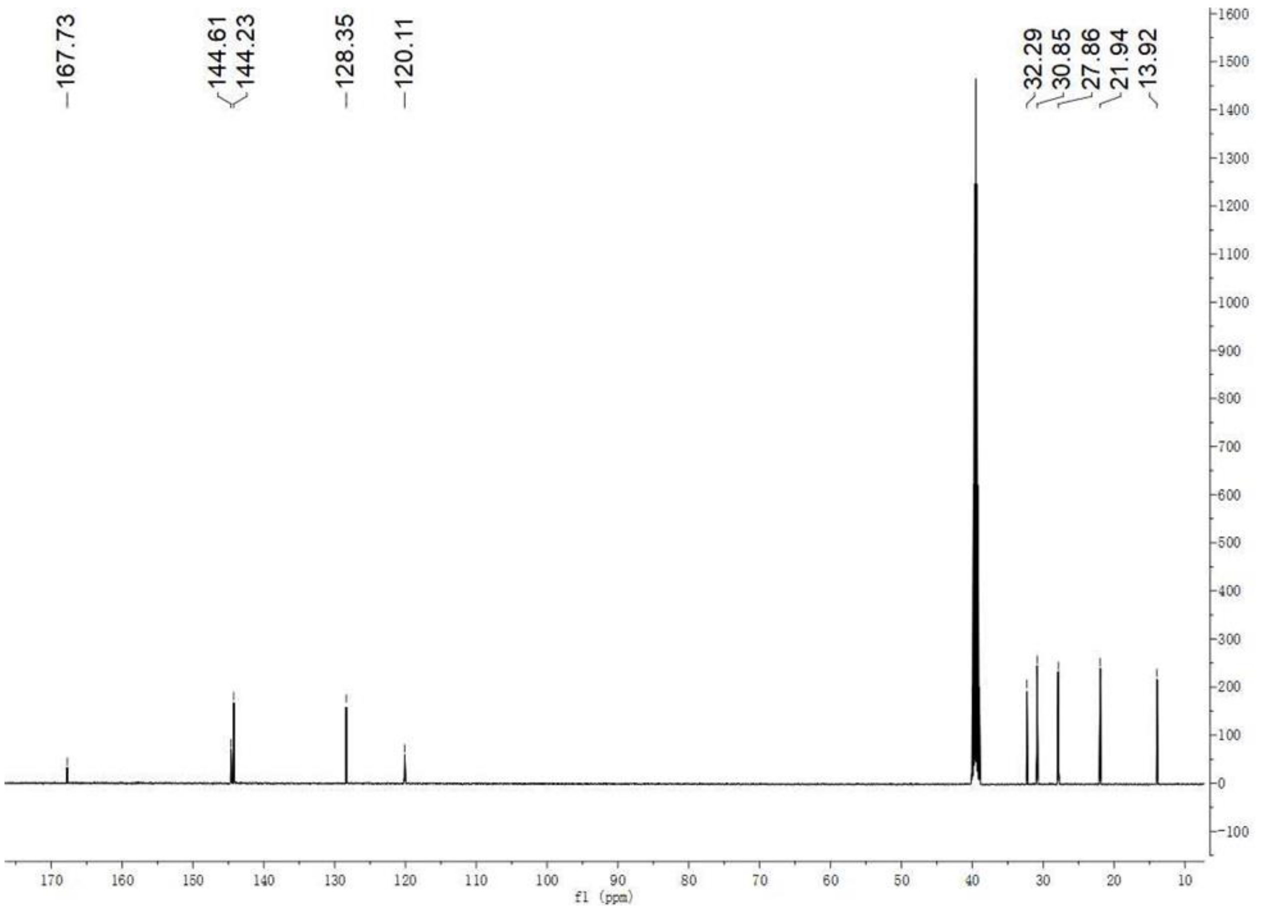

Fig. S2 ^{13}C NMR (125 MHz, DMSO-d_6) spectrum of DDA.

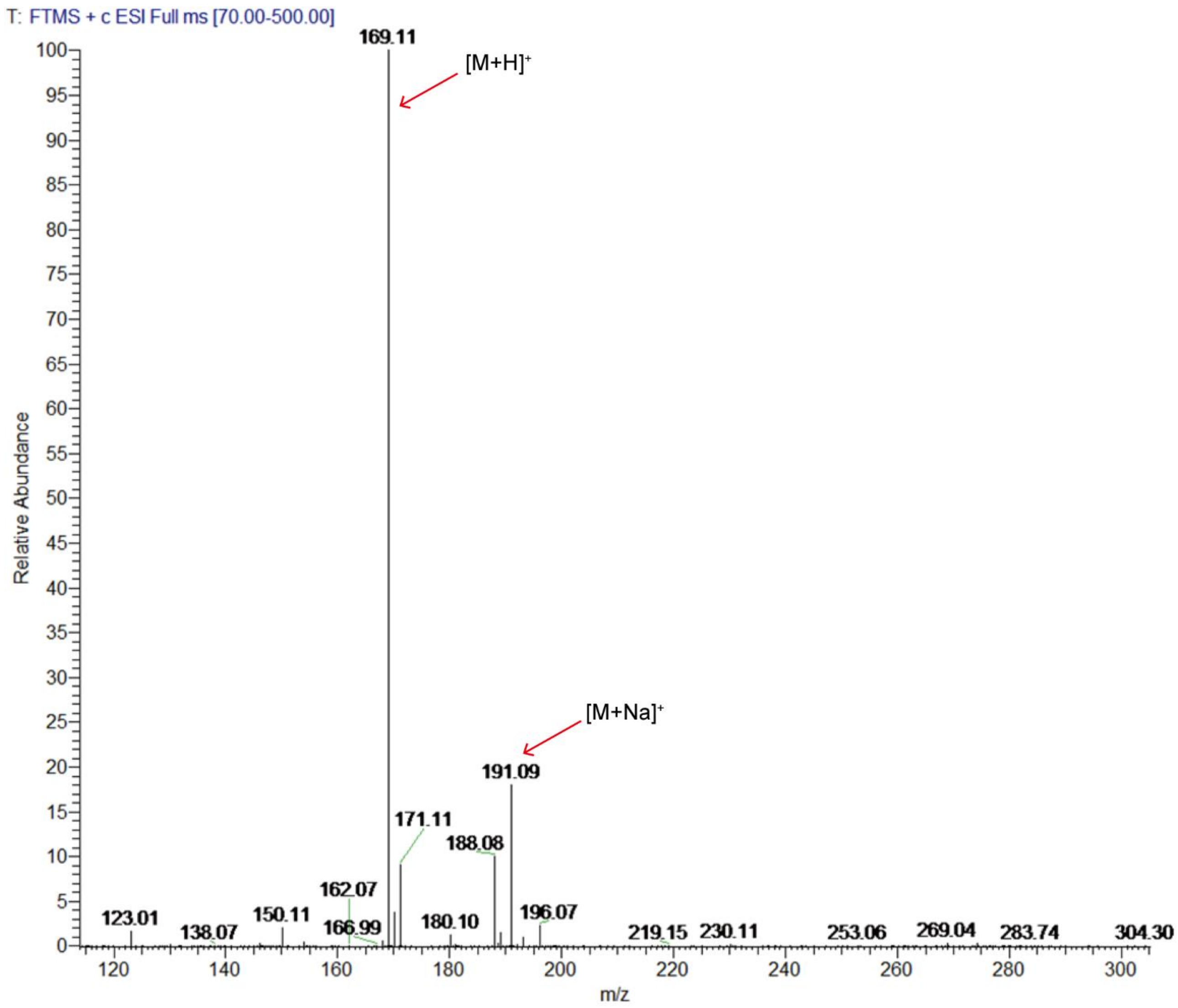

Fig. S3 ESIMS spectrum of DDA.

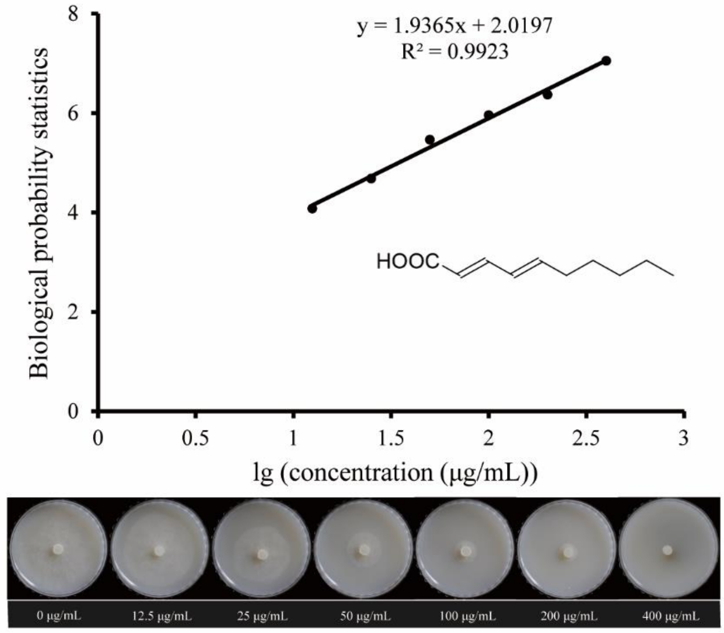

Fig. S4 Toxicity regression equation of DDA on *P. nicotianae*. The x-axis represents the lg value of concentrations, whereas the y-axis represents the biological probability statistics corresponding to the inhibition rate.

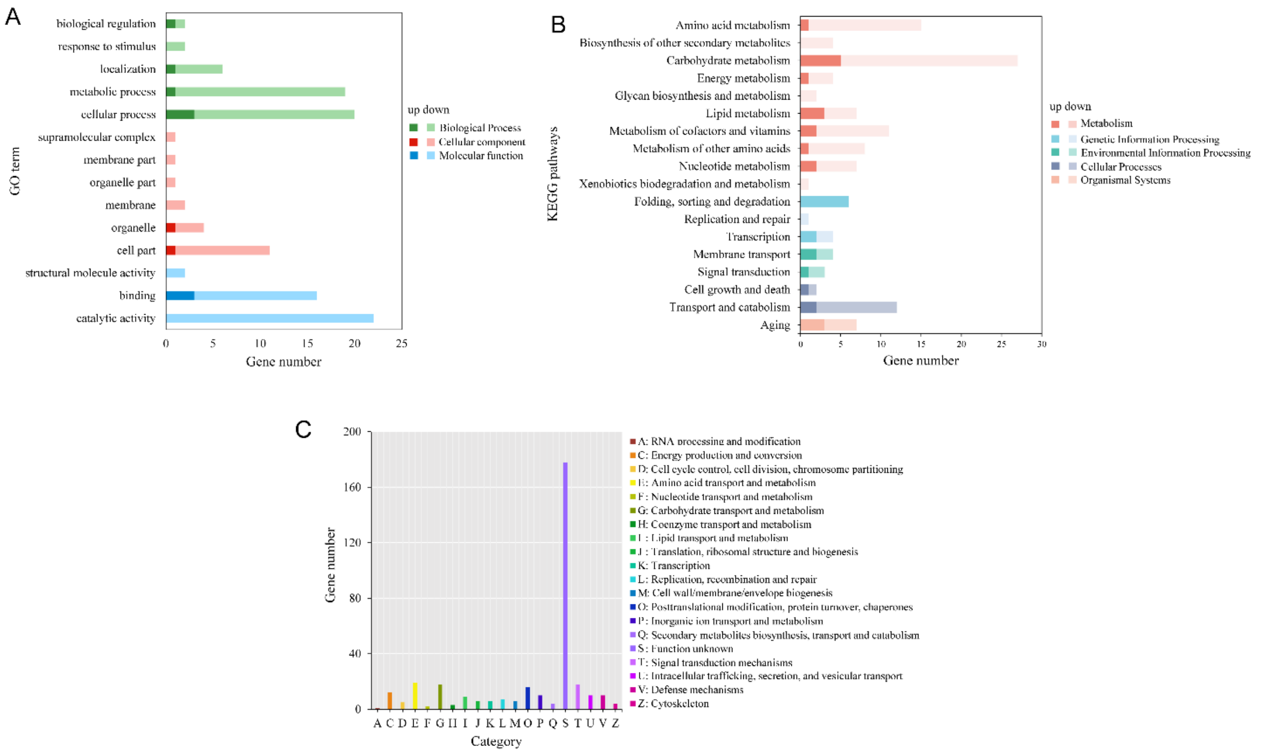

Fig. S5 Classification of DEGs. (A) GO classification of DEGs. The x-axis represents the number of genes, whereas the y-axis represents the DEGs' respective categories. (B) KEGG classification of DEGs. The x-axis represents the number of genes, whereas the y-axis represents the DEGs' respective categories. (C) COG classification of DEGs. The x-axis represents groups (shown on right of histogram), and y-axis represents gene number.

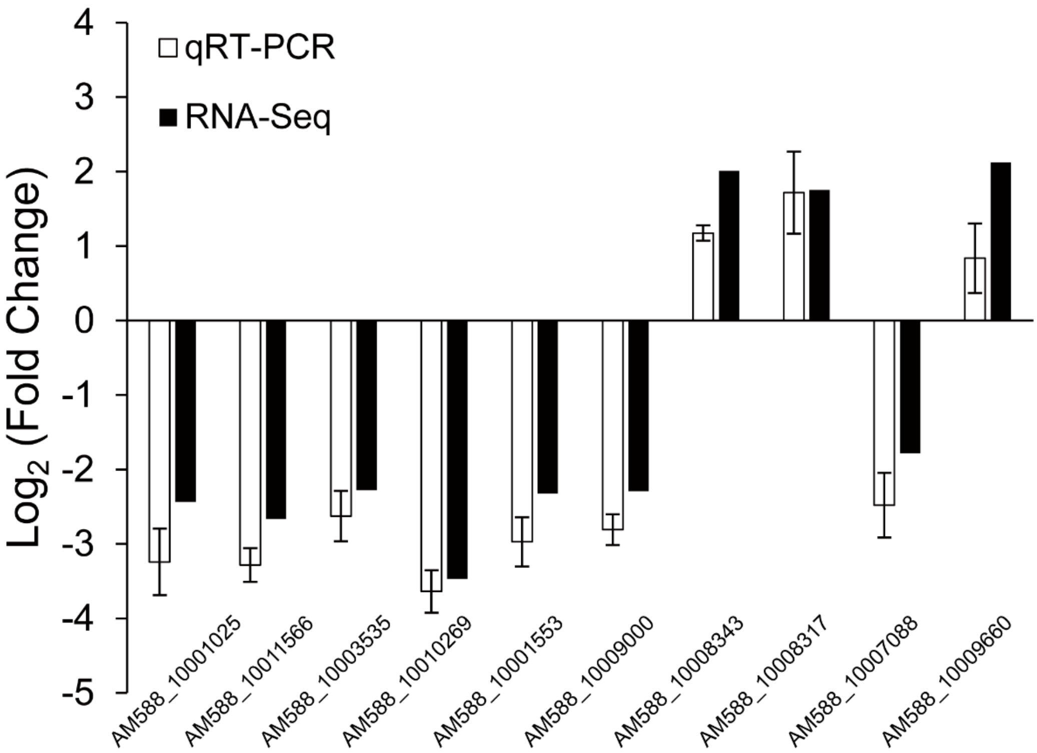

Fig. S6 Validation of RNA-Seq results using qRT-PCR. A total of 10 DEGs were randomly selected for qRT-PCR from RNA-Seq genes.

Table S1 Inhibition rates of Fr.1-7 (0.5 mg/mL) isolated from co-culture fermentation broth on *P. nicotianae*.

Components	Inhibition rate (%)
Fr. 1	7.870±0.65g
Fr. 2	30.71±0.37d
Fr. 3	90.45±0.06a
Fr. 4	59.14±0.10b
Fr. 5	34.27±0.13c
Fr. 6	14.08±0.16f
Fr. 7	15.73±0.65e

Table S2 Summary of RNA-seq reads in control (CK) and treatment (T) groups of *P. nicotianae*.

Sample	Raw reads	Clean reads	Q30(%)	GC content (%)	Total mapped
T_2	44383636	44077620	96.16	57.64	42165905(95.66%)
T_3	50406106	49927488	94.86	57.29	47450039(95.04%)
T_1	45603582	45163564	96	57.14	42593591(94.31%)
C_3	43777314	43470676	96	57.1	41028729(94.38%)
C_1	43588358	43273710	95.94	57.18	40900466(94.52%)
C_2	46342188	45842266	94.53	57.28	43580289(95.07%)

Table S3 DEGs in GO enrichment analysis.

GO ID	Term	Description	Ratio_in_study	Ratio_in_pop	Pvalue
GO:0006801	BP	superoxide metabolic process	2/42	2/1208	0.001181025
GO:0072593	BP	reactive oxygen species metabolic process	2/42	2/1208	0.001181025
GO:0016491	MF	oxidoreductase activity	8/42	64/1208	0.001121881
GO:0016614	MF	oxidoreductase activity, acting on CH-OH group of donors	3/42	7/1208	0.001242845
GO:0016616	MF	oxidoreductase activity, acting on the CH-OH group of donors, NAD or NADP as acceptor	3/42	7/1208	0.001242845
GO:0046394	BP	carboxylic acid biosynthetic process	4/42	18/1208	0.00271468
GO:0016053	BP	organic acid biosynthetic process	4/42	18/1208	0.00271468
GO:0043436	BP	oxoacid metabolic process	6/42	55/1208	0.009941816
GO:0006082	BP	organic acid metabolic process	6/42	55/1208	0.009941816
GO:0019752	BP	carboxylic acid metabolic process	6/42	55/1208	0.009941816
GO:0044283	BP	small molecule biosynthetic process	4/42	29/1208	0.015945196
GO:1901607	BP	alpha-amino acid biosynthetic process	2/42	7/1208	0.022189303
GO:1901566	BP	organonitrogen compound biosynthetic process	6/42	69/1208	0.028533985
GO:0006022	BP	aminoglycan metabolic process	1/42	1/1208	0.034768212
GO:0006023	BP	aminoglycan biosynthetic process	1/42	1/1208	0.034768212
GO:0006024	BP	glycosaminoglycan biosynthetic process	1/42	1/1208	0.034768212
GO:0097164	BP	ammonium ion metabolic process	1/42	1/1208	0.034768212
GO:0046398	BP	UDP-glucuronate metabolic process	1/42	1/1208	0.034768212
GO:0006102	BP	isocitrate metabolic process	1/42	1/1208	0.034768212
GO:0009102	BP	biotin biosynthetic process	1/42	1/1208	0.034768212

Table S4 DEGs in KEGG enrichment analysis.

Pathway id	Category	Description	Ratio_in_study	Ratio_in_pop	Pvalue
map00620	Metabolism	Pyruvate metabolism	9/133	44/4075	8.617E-06
map00232	Metabolism	Caffeine metabolism	3/133	3/4075	3.40121E-05
map04213	Organismal Systems	Longevity regulating pathway - multiple species	7/133	42/4075	0.000349777
map00640	Metabolism	Propanoate metabolism	6/133	32/4075	0.000486182
map00280	Metabolism	Valine, leucine and isoleucine degradation	6/133	40/4075	0.001662149
map00630	Metabolism	Glyoxylate and dicarboxylate metabolism	5/133	31/4075	0.002946309
map00410	Metabolism	beta-Alanine metabolism	4/133	27/4075	0.010622465
map00780	Metabolism	Biotin metabolism	2/133	5/4075	0.009910824
map04146	Cellular Processes	Peroxisome	7/133	71/4075	0.007863174
map00230	Metabolism	Purine metabolism	7/133	73/4075	0.009129012
map00520	Metabolism	Amino sugar and nucleotide sugar metabolism	4/133	32/4075	0.019203373
map00260	Metabolism	Glycine, serine and threonine metabolism	5/133	48/4075	0.019095496
map00790	Metabolism	Folate biosynthesis	3/133	22/4075	0.033258711
map00290	Metabolism	Valine, leucine and isoleucine biosynthesis	2/133	9/4075	0.032749217
map00254	Metabolism	Aflatoxin biosynthesis	1/133	1/4075	0.032638037
map00791	Metabolism	Atrazine degradation	1/133	1/4075	0.032638037
map00380	Metabolism	Tryptophan metabolism	3/133	25/4075	0.046293551
map00680	Metabolism	Methane metabolism	3/133	27/4075	0.056183914
map00770	Metabolism	Pantothenate and CoA biosynthesis	2/133	14/4075	0.074471747
map02010	Environmental Information Processing	ABC transporters	4/133	52/4075	0.088244028

Table S5 Summary of major genes and metabolites related to oxidative stress affected in *Phytophthora nicotianae* exposed to DDA compared to untreated groups.

gene/metab ID	KEGG pathway/compound	Enzyme/Metobolite	Fold change (T/C)	P-value
Peroxisome				
AM588_10003508	K04564	SOD2; superoxide dismutase, Fe-Mn family [EC:1.15.1.1]	0.386	7.89E-18
AM588_10005720	K04564	SOD2; superoxide dismutase, Fe-Mn family [EC:1.15.1.1]	0.488	9.5692E-09
AM588_10010269	K00031	IDH1, IDH2, icd; isocitrate dehydrogenase [EC:1.1.1.42]	0.09	6.2544E-09
AM588_10011662	K00428	E1.11.1.5 cytochrome c peroxidase [EC:1.11.1.5]	0.442	3.0325E-11
AM588_10009488	K03781	katE, CAT, catB, srpA; catalase [EC:1.11.1.6]	0.121	4.4312E-33
AM588_10004917	K03781	katE, CAT, catB, srpA; catalase [EC:1.11.1.6]	0.462	3.5350E-13
AM588_10009585	K00106	XDH; xanthine dehydrogenase/oxidase [EC:1.17.1.4 1.17.3.2]	0.365	9.4735E-12
AM588_10007088	K13348	MPV17; protein Mpv17	0.291	9.3645E-07
Glyoxylate and dicarboxylate metabolism				
AM588_10001566	K00122	FDH; formate dehydrogenase [EC:1.17.1.9]	0.403	3.6988E-05
Pentose and glucuronate interconversions				
AM588_10009542	K00012	UGDH, ugd; UDPglucose 6-dehydrogenase [EC:1.1.1.22]	0.415	5.8606E-14
Not Included in Pathway or Brite				
AM588_10000552	K23516	XAN1; xanthine dioxygenase [EC:1.14.11.48]	0.279	2.4444E-14
Oxidative phosphorylation				
metab_34	C00042	succinic acid	0.9547	0.00007464

Table S6 Summary of major genes and metabolites related to membrane homeostasis affected in *Phytophthora nicotianae* exposed to DDA compared to untreated groups.

gene/metab ID	KEGG pathway/compound	Enzyme	Fold change (T/C)	P-value
Glycerophospholipid metabolism				
AM588_10009295	K00968	PCYT1; choline-phosphate cytidylyltransferase [EC:2.7.7.15]	0.498	0.003759158
AM588_10008936	K08730	PTDSS2; phosphatidylserine synthase 2 [EC:2.7.8.29]	0.412	6.19882E-09
AM588_10008053	K14674	TGL4; TAG lipase / steryl ester hydrolase / phospholipase A2 / LPA acyltransferase [EC:3.1.1.3 3.1.1.13 3.1.1.4 2.3.1.51]	2.015	9.05846E-09
Ether lipid metabolism				
AM588_10008053	K14674	TGL4; TAG lipase / steryl ester hydrolase / phospholipase A2 / LPA acyltransferase [EC:3.1.1.3 3.1.1.13 3.1.1.4 2.3.1.51]	2.015	9.05846E-09
Phosphatidylinositol signaling system				
AM588_10007265	K01110	PTEN; phosphatidylinositol-3,4,5-trisphosphate 3-phosphatase and dual-specificity protein phosphatase PTEN [EC:3.1.3.16 3.1.3.48 3.1.3.67]	2.822	9.1564E-05
Inositol phosphate metabolism				
AM588_10007228	K00140	mmsA, iolA, ALDH6A1; malonate-semialdehyde dehydrogenase (acetylating) / methylmalonate-semialdehyde dehydrogenase [EC:1.2.1.18 1.2.1.27]	0.476	6.9105E-07
AM588_10007265	K01110	PTEN; phosphatidylinositol-3,4,5-trisphosphate 3-phosphatase and dual-specificity protein phosphatase PTEN [EC:3.1.3.16 3.1.3.48 3.1.3.67]	2.822	9.1564E-05
Sphingolipid metabolism				
AM588_10005926	K12349	ASAH2; neutral ceramidase [EC:3.5.1.23]	0.466	8.39145E-06
MAPK signaling pathway				
AM588_10003838	K17686	copA, ctpA, ATP7; P-type Cu+ transporter [EC:7.2.2.8]	0.302	2.60263E-16
cAMP signaling pathway				
AM588_10002209	K01539	ATP1A; sodium/potassium-transporting ATPase subunit alpha [EC:7.2.2.13]	0.183	9.39322E-09
ABC transporters				
AM588_10010234	K05658	ABCB1, CD243; ATP-binding cassette, subfamily B (MDR/TAP), member 1 [EC:7.6.2.2]	0.354	5.65804E-16

AM588_10005733	K05668	ABCC5; ATP-binding cassette, subfamily C (CFTR/MRP), member 5	0.48	0.000126916
Cysteine and methionine metabolism				
AM588_10002658	K00826	ilvE; Phospholipid-transporting ATPase 3	0.359	8.82895E-12
AM588_10002199	ko00001	DRS2, ATP8A; phospholipid-transporting ATPase [EC:7.6.2.1]	0.459	1.06136E-13
Fatty acid biosynthesis				
metab_25	C00383; C04025	malonic acid	0.977	0.001656
metab_75	C06424	myristic acid	1.026	0.0003191
metab_90	C08362	palmitoleic acid	1.050	0.0000596

Table S7 Summary of major genes and metabolites related to energy and substance metabolism affected in *Phytophthora nicotianae* exposed to 4-decadienoic acid compared to untreated groups.

ID	KEGG pathway	Enzyme	Fold change (T/C)	P-value
Pyruvate metabolism				
AM588_10011566	K01638	aceB, glcB; malate synthase [EC:2.3.3.9]	0.157	2.2086E-08
AM588_10008694	K00029	maeB; malate dehydrogenase (oxaloacetate-decarboxylating) (NADP+) [EC:1.1.1.40]	0.29	1.43652E-05
AM588_10001650	K01958	PC, pyc; pyruvate carboxylase [EC:6.4.1.1]	0.225	5.13136E-16
AM588_10005144	K11262	ACACA; acetyl-CoA carboxylase / biotin carboxylase 1 [EC:6.4.1.2 6.3.4.14 2.1.3.15]	0.219	2.08308E-06
AM588_10006813	K01069	gloB, gloC, HAGH; hydroxyacylglutathione hydrolase [EC:3.1.2.6]	0.476	2.90479E-07
AM588_10003118	K01069	gloB, gloC, HAGH; hydroxyacylglutathione hydrolase [EC:3.1.2.6]	0.476	2.90479E-07
AM588_10000957	K01006	ppdK; pyruvate, orthophosphate dikinase [EC:2.7.9.1]	2.217	1.41391E-07
AM588_10009682	K01895	ACSS1_2, acs; acetyl-CoA synthetase [EC:6.2.1.1]	2.361	0.000281932
AM588_10009195	K00128	ALDH; aldehyde dehydrogenase (NAD+) [EC:1.2.1.3]	2.227	7.93902E-08
metab_34	C00042	succinic acid	0.9547	0.00007464
Propanoate metabolism				
AM588_10005144	K11262	ACACA; acetyl-CoA carboxylase / biotin carboxylase 1 [EC:6.4.1.2 6.3.4.14 2.1.3.15]	0.219	2.08308E-06
AM588_10007228	K00140	mmsA, iolA, ALDH6A1; malonate-semialdehyde dehydrogenase (acetylating) / methylmalonate-semialdehyde dehydrogenase [EC:1.2.1.18 1.2.1.27]	0.476	6.9105E-07
AM588_10001553	K00166	BCKDHA, bkdA1; 2-oxoisovalerate dehydrogenase E1 component alpha subunit [EC:1.2.4.4]	0.2	5.66989E-24
AM588_10011688	K00167	BCKDHB, bkdA; 2-oxoisovalerate dehydrogenase E1 component beta subunit [EC:1.2.4.4]	0.237	2.75499E-25
AM588_10011686	K09699	DBT, bkdB; 2-oxoisovalerate dehydrogenase E2 component (dihydrolipoyl transacylase) [EC:2.3.1.168]	0.114	2.38452E-46
AM588_10009682	K01895	ACSS1_2, acs; acetyl-CoA synthetase [EC:6.2.1.1]	2.361	0.000281932
metab_34	C00042	succinic acid	0.9547	0.00007464
Valine, leucine and isoleucine degradation				
AM588_10002658	K00826	ilvE; branched-chain amino acid aminotransferase [EC:2.6.1.42]	0.359	8.82895E-12
AM588_10001553	K00166	BCKDHA, bkdA1; 2-oxoisovalerate dehydrogenase E1 component alpha subunit [EC:1.2.4.4]	0.2	5.66989E-24
AM588_10011688	K00167	BCKDHB, bkdA; 2-oxoisovalerate dehydrogenase E1 component beta subunit [EC:1.2.4.4]	0.237	2.75499E-25

AM588_10011686	K09699	DBT, bkdB; 2-oxoisovalerate dehydrogenase E2 component (dihydrolipoyl transacylase) [EC:2.3.1.168]	0.114	2.38452E-46
AM588_10007228	K00140	mmsA, iolA, ALDH6A1; malonate-semialdehyde dehydrogenase (acetylating) / methylmalonate-semialdehyde dehydrogenase [EC:1.2.1.18 1.2.1.27]	0.476	6.9105E-07
AM588_10009195	K00128	ALDH; aldehyde dehydrogenase (NAD+) [EC:1.2.1.3]	2.227	7.93902E-08
Glyoxylate and dicarboxylate metabolism				
AM588_10011566	K01638	aceB, glcB; malate synthase [EC:2.3.3.9]	0.157	2.20868E-08
AM588_10001566	K00122	FDH; formate dehydrogenase [EC:1.17.1.9]	0.403	3.69881E-05
AM588_10009488	K03781	katE, CAT, catB, srpA; catalase [EC:1.11.1.6]	0.121	4.43119E-33
AM588_10004917	K03781	katE, CAT, catB, srpA; catalase [EC:1.11.1.6]	0.462	3.53501E-13
AM588_10009682	K01895	ACSS1_2, acs; acetyl-CoA synthetase [EC:6.2.1.1]	2.361	0.000281932
metab_18	C00209	oxalic acid	0.9810	0.0001273
metab_34	C00042	succinic acid	0.9547	0.00007464
metab_35	C00258	glyceric acid	1.0586	0.00004957
metab_72	C00158	citric acid	0.9756	0.01182
Purine metabolism				
AM588_10008008	K18437	PDE8; high affinity cAMP-specific and IBMX-insensitive 3',5'-cyclic phosphodiesterase 8 [EC:3.1.4.53]	2.227	1.77785E-11
AM588_10011663	K13298	PDE11; dual 3',5'-cyclic-AMP and -GMP phosphodiesterase 11 [EC:3.1.4.17 3.1.4.35]	0.402	5.60601E-05
AM588_10009585	K00106	XDH; xanthine dehydrogenase/oxidase [EC:1.17.1.4 1.17.3.2]	0.365	9.47353E-12
AM588_10003535	K00365	uaZ; urate oxidase [EC:1.7.3.3]	0.206	8.6442E-07
AM588_10004044	K00365	uaZ; urate oxidase [EC:1.7.3.3]	0.367	0.001676289
AM588_10009250	K01427	URE; urease [EC:3.5.1.5]	0.417	1.99581E-10
AM588_10004145	K18550	ISN1; IMP and pyridine-specific 5'-nucleotidase [EC:3.1.3.99 3.1.3.-]	2.246	1.20187E-08
metab_18	C00209	oxalic acid	0.9810	0.0001273
metab_130	C00294	inosine	1.0222	0.002856
metab_133	C00212	adenosine	1.0247	0.002617
Amino sugar and nucleotide sugar metabolism				
AM588_10009542	K00012	UGDH, ugd; UDPglucose 6-dehydrogenase [EC:1.1.1.22]	0.415	5.8606E-14
AM588_10003702	K08679	GAE, cap1J; UDP-glucuronate 4-epimerase [EC:5.1.3.6]	0.391	2.66849E-09
AM588_10003703	K08679	GAE, cap1J; UDP-glucuronate 4-epimerase [EC:5.1.3.6]	0.348	2.37677E-10
AM588_10009818	K05305	FUK; fucokinase [EC:2.7.1.52]	0.379	4.21562E-08
Glycine, serine and threonine metabolism				
AM588_10005293	K00108	betA, CHDH; choline dehydrogenase [EC:1.1.99.1]	0.394	5.49046E-17

AM588_10003795	K01754	ilvA, tdcB; threonine dehydratase [EC:4.3.1.19]	0.199	0.000246012
AM588_10000644	K01733	thrC; threonine synthase [EC:4.2.3.1]	0.474	1.75936E-12
AM588_10001269	K00276	AOC3, AOC2, tynA; primary-amine oxidase [EC:1.4.3.21]	0.268	6.30478E-25
AM588_10000329	K00276	AOC3, AOC2, tynA; primary-amine oxidase [EC:1.4.3.21]	0.203	9.70438E-17
metab_35	C00258	glyceric acid	1.0586	0.00004957
metab_56	C00097	L-cysteine	1.0323	0.00988
beta-Alanine metabolism				
AM588_10007228	K00140	mmsA, iolA, ALDH6A1; malonate-semialdehyde dehydrogenase (acetylating) / methylmalonate-semialdehyde dehydrogenase [EC:1.2.1.18 1.2.1.27]	0.476	6.9105E-07
AM588_10001269	K00276	AOC3, AOC2, tynA; primary-amine oxidase [EC:1.4.3.21]	0.268	6.30478E-25
AM588_10000329	K00276	AOC3, AOC2, tynA; primary-amine oxidase [EC:1.4.3.21]	0.203	9.70438E-17
AM588_10009195	K00128	ALDH; aldehyde dehydrogenase (NAD+) [EC:1.2.1.3]	2.227	7.93902E-08
metab_25	C00383; C04025	malonic acid	0.9771	0.001656
metab_81	C00135	L-histidine	0.9249	0.002602
Citrate cycle (TCA cycle)				
AM588_10001650	K01958	PC, pyc; pyruvate carboxylase [EC:6.4.1.1]	0.225	5.13136E-16
AM588_10010269	K00031	IDH1, IDH2, icd; isocitrate dehydrogenase [EC:1.1.1.42]	0.09	6.25442E-09
metab_34	C00042	succinic acid	0.9547	0.00007464
metab_72	C00158	citric acid	0.9756	0.01182
Glycolysis / Gluconeogenesis				
AM588_10001791	K03841	FBP, fbp; fructose-1,6-bisphosphatase I [EC:3.1.3.11]	0.5	2.84237E-05
AM588_10009682	K01895	ACSS1_2, acs; acetyl-CoA synthetase [EC:6.2.1.1]	2.361	0.000281932
AM588_10009195	K00128	ALDH; aldehyde dehydrogenase (NAD+) [EC:1.2.1.3]	2.227	7.93902E-08
Sulfur relay system				
metab_56	C00097	L-cysteine	1.0323	0.00988
Taurine and hypotaurine metabolism				
metab_56	C00097	L-cysteine	1.0323	0.00988
metab_111	C00519	hypotaurine	1.0202	0.001325
Alanine, aspartate and glutamate metabolism				
metab_34	C00042	succinic acid	0.9547	0.00007464
metab_72	C00158	citric acid	0.9756	0.01182

Table S8 Metabolite information detected by GC-MS. (Table shown in separate file)

Table S9 Information on the screening of differential metabolites.

Compund name	VIP	P-value
Anisomycin	3.186163242	4.11E-06
benzoylformic acid	2.434360655	0.0002796
D-malic acid	1.927533432	3.36E-05
azelaic acid	1.883430402	0.0007211
D-lyxose	1.878009001	4.13E-08
citraconic acid	1.838662792	6.27E-06
L-histidine	1.798102568	0.002602
1-hydroxy-2-naphthoic acid	1.787644994	0.002174
succinic acid	1.769248893	7.46E-05
5,6-dihydro-5-methyluracil	1.735801589	0.0001919
1-octen-3-ol	1.693120974	5.38E-05
carbazole	1.682713985	0.02028
threonic acid	1.675826094	0.02615
glyceric acid	1.608759576	4.96E-05
palmitoleic acid	1.55628376	5.96E-05
cellotetraose	1.547871685	1.93E-05
N-acetyl-5-hydroxytryptamine	1.504572154	0.01163
3-methyl-L-histidine	1.464685701	0.0003134
fructose-1,6-diphosphate	1.448880571	0.0008163
N-carbobenzoxy-L-leucine	1.427835767	3.71E-06
valeramide	1.391066173	0.0016
psicose	1.378342751	0.0004265
phlorobenzophenone	1.370883676	0.005054
methyl-beta-D-galactopyranoside	1.34536491	0.002123
L-methionine	1.305139825	0.01188
myristic acid	1.266978611	0.0003191
biphenyl	1.212111803	9.26E-05
oxalic acid	1.205349879	0.0001273
L-cysteine	1.201210553	0.00988
malonic acid	1.193650618	0.001656
6-hydroxynicotinic acid	1.190298436	0.02017
3-hydroxyflavone	1.188029465	0.003679
adenosine	1.179575819	0.002617
1,2,4,5-tetramethylbenzene	1.178982375	0.0001123
glycylproline	1.170509783	0.001197
citric acid	1.169674365	0.01182
benzyl thiocyanate	1.153475029	0.002465
eicosane	1.15152511	0.0003355
L- (+) lactic acid	1.149341351	0.002446
hexadecene	1.144785182	0.04527
D-Ala-D-Ala2	1.140935702	0.001412
D (+) galactose	1.13646214	0.0002324

ribulose-5-phosphate	1.131302129	0.0009572
4-hydroxyquinoline	1.121085914	0.008567
inosine	1.099920612	0.002856
adrenaline	1.045192928	0.0009525
linoleic acid	1.04021247	0.003583
1-methylhydantoin	1.039355146	0.003829
D-glucose-6-phosphate	1.03502878	0.006706
12a-Hydroxy-9-Demethylmunduserone-8-Carboxylic Acid	1.033167006	0.0004259
2-aminophenol	1.023522715	0.02912
3-(methylthio)-propylamine	1.021915311	0.0007561
hypotaurine	1.008648234	0.001325
3-hydroxypyridine	1.007521821	0.001174

Table S10 DEMs in KEGG enrichment analysis.

Pathway Description	Pathway_ID	Pvalue
Glyoxylate and dicarboxylate metabolism	map00630	0.0006
Aminoacyl-tRNA biosynthesis	map00970	0.0043
Fatty acid biosynthesis	map00061	0.0058
Citrate cycle (TCA cycle)	map00020	0.0071
Taurine and hypotaurine metabolism	map00430	0.0085
Alanine, aspartate and glutamate metabolism	map00250	0.0136
beta-Alanine metabolism	map00410	0.0176
Sulfur metabolism	map00920	0.0187
Purine metabolism	map00230	0.0223
Butanoate metabolism	map00650	0.0294
Histidine metabolism	map00340	0.0362
Glycine, serine and threonine metabolism	map00260	0.0405
Nicotinate and nicotinamide metabolism	map00760	0.0481

Table S11 Primers used for qRT-PCR analysis

gene id	FORWARD	REVERSE
AM588_10001025	GCGGACCACGAGAAGAAGTACAAC	CGGCTCCTGCTTGACGATGAAC
AM588_10011566	GCACCCGCAGACAGGAAAGAAG	GCACATGAGCCTCGTCCAGATG
AM588_10003535	GTGCCTACTATGACGGTGACAATGG	TCTGGGTGCTTCGTGACAAAGTG
AM588_10010269	GGCTCAAGAAGATGTGGCTCAGTC	GCGTGACGACCGATGATGATAGG
AM588_10001553	GATCCAAGAACCTCAACCACCAGAC	ACGGTCCTCCTTTGCCAACTTAAAC
AM588_10009000	AGGGCAGGCAAACGCTATGAAC	TAGGCTTCCGAGGCATCTGAGAC
AM588_10008343	GATGCCAGAGAACACGCCGTAC	CGTCGTCCACATCCAATAGGAAGTC
AM588_10008317	AATGGAAGGCGACTGGTGGAATG	CGATACACGAGGCACAGCAAGG
AM588_10007088	CCGTGCTCTTTGGACTTGGAGATC	CGCTGATGAGATGACCCTCTTTGAC
AM588_10009660	TTATCATTCTCCACGCCGTCACATC	CTCGCCAAGCCTTCACCTTATCG
β-Actin	ACTGCACGTTCCAGACGATC	CCACCACCTTGATCTTCATG
